# Revisiting Intercalation Anode Materials for Potassium-Ion Batteries

**DOI:** 10.3390/ma18010190

**Published:** 2025-01-04

**Authors:** María José Piernas-Muñoz, Maider Zarrabeitia

**Affiliations:** 1 Inorganic Chemistry Department, Faculty of Chemistry, University of Murcia, Campus Universitario St. 5, 30100 Murcia, Spain; 2 Helmholtz Institute Ulm (HIU), Helmholtzstraße 11, 89081 Ulm, Germany; maider.ipina@kit.edu; 3 Karlsruhe Institute of Technology (KIT), P.O. Box 3640, 76021 Karlsruhe, Germany

**Keywords:** Potassium ion, batteries, rechargeable, intercalation, carbonaceous materials, graphite, hard carbon, soft carbon, oxides, anode, materials

## Abstract

Potassium-ion batteries (KIBs) have attracted significant attention in recent years as a result of the urgent necessity to develop sustainable, low-cost batteries based on non-critical raw materials that are competitive with market-available lithium-ion batteries. KIBs are excellent candidates, as they offer the possibility of providing high power and energy densities due to their faster K^+^ diffusion and very close reduction potential compared with Li^+^/Li. However, research on KIBs is still in its infancy, and hence, more investigation is required both at the materials level and at the device level. In this work, we focus on recent strategies to enhance the electrochemical properties of intercalation anode materials, i.e., carbon-, titanium-, and vanadium-based compounds. Hitherto, the most promising anode materials are those carbon-based, such as graphite, soft, or hard carbon, each with its advantages and disadvantages. Although a wide variety of strategies have been reported with excellent results, there is still a need to improve the standardization of the best carbon properties, electrode formulation, and electrolyte composition, given the impossibility of a direct comparison. Therefore, additional effort should be made to understand what are the crucial carbon parameters to develop a reference electrode and electrolyte formulation to further boost their performance and move a step forward in the commercialization of KIBs.

## 1. Introduction

The current ever-growing energy demand, along with the environmental pollution caused by fossil fuels, makes necessary a shift toward cleaner, sustainable energy sources. However, the associated intermittency of renewable energies requires the utilization of energy-storage devices, being rechargeable batteries, specifically lithium-ion batteries (LIBs), one of the most popular choices [1,2]. In addition to large-scale applications, such as grid-scale, LIBs dominate the market of portable electronic devices due to their high energy density and long cycle life, and they also power EVs (electric vehicles), which globally reached nearly 14 million sales in 2023 [3]. Nonetheless, this successful technology presents certain limitations. LIB production is limited by the scarce, costly, and unevenly distributed lithium resources [4,5,6], and the prospects for their recycling [7] are not yet encouraging enough. Emerging alternatives to LIBs based on abundant (hence, more economical) alkali metals available worldwide that are now under exhaustive research are sodium-ion batteries (NIBs) [8,9,10,11,12,13,14] and KIBs [15,16,17,18,19,20,21]. Sodium (2.4 wt.%) and potassium (2.1 wt.%) are, respectively, the sixth and the eighth most abundant elements on the Earth’s crust, after oxygen (46.1 wt.%), silicon (28.2 wt.%), aluminum (8.23 wt.%), iron (5.63 wt.%), and calcium (4.15 wt.%) [22]. Both NIBs and KIBs follow the same working principle as LIBs, where the corresponding alkali ions “rock” back and forth between the electrodes that build up the battery [23]. Furthermore, given their similar physicochemical properties, most of the knowledge acquired over the years in LIBs, such as the types of electrode materials and electrolytes used as well as the manufacturing processes, could, in principle, be transferred to NIBs and KIBs, positioning them as drop-in technologies [19,24]. Regrettably, this is not so straightforward, since sodium and potassium have larger ionic radii than lithium (see Table 1) and hence, the host electrodes in NIBs and KIBs must provide sites big enough to accommodate them within their structure. On the other hand, unlike LIBs, NIBs and KIBs can benefit from the use of aluminum foil as the current collector at the anode, since Na and K do not alloy with aluminum at low voltages. Replacing copper with aluminum not only translates into battery cost [15] and weight reductions, opening the possibility of bipolar stacking [17], but also into greater safety, as batteries can be transported and stored fully discharged [25]. Nonetheless, despite the aforementioned similarities, KIBs possess three valuable advantages over NIBs (please see Table 1):

(1) K^+^ has the smallest Stokes (or solvated) radius compared with Li^+^ and Na^+^ due to its weaker Lewis acidity, resulting in faster diffusion of K^+^ in either aqueous or non-aqueous electrolytes, and hence, it would enable enhanced power capability, which is extremely beneficial for the fast charging and discharging requirement that the market demands lately [18,25].

(2) The standard reduction potential K^+^/K (vs. SHE) is lower than that of Na^+^/Na and closer to that of Li^+^/Li, guaranteeing a higher cell operating voltage and, therefore, theoretically surpassing the constrained energy density of NIBs [15,16,17]. Indeed, as shown in Table 1, K^+^/K exhibits a lower potential than that of Li^+^/Li in PC (propylene carbonate) [26] and EC:DEC (ethylene carbonate: diethyl carbonate), encouraging the possibility that KIBs may achieve even higher cell voltages than LIBs as long as the cathodes in KIBs display the same voltages as their LIBs analogues [15].

(3) Furthermore, unlike Na^+^, K^+^ is intercalated into commercially available graphite anode electrodes, as we will further detail in the subsequent paragraphs. This represents a step forward toward the potential industrial production of KIBs truly being a “drop-in technology”, which can be quickly transferred to standard LIB production [19].

**Table 1:** Comparison of some physicochemical, electrochemical, and economic properties of Li, Na, and K.

Properties	Li	Na	K
Atomic mass, u	6.941	22.989	39.098
Melting point, °C	180.5	97.7	63.4
Atomic radius, pm	145	180	220
Ionic radius, Å [27]	0.76	1.02	1.38
Stokes radius in water, Å [28]	2.38	1.84	1.25
Stokes radius in PC, Å [29]	4.8	4.6	3.6
Voltage (A^+^/A) vs. SHE, ^1^ V [30]	−3.04	−2.71	−2.93
Voltage (A^+^/A) vs. Li^+^/Li in PC, V [30]	0	0.23	−0.09
Voltage (A^+^/A) vs. Li^+^/Li in EC:DEC, V [31]	0	**-**	−0.15
Theoretical capacity of graphite, mAh g^−1^ [32]	372	111.7 ^2^	279
Crust abundance, mass % [22]	0.0017	2.4	2.1
Distribution [20]	70% S. Am. ^3^	Global	Global
Cost of carbonate [33], ^4^ USD ton^−1^	13,860	350	1540

^1^ A = Li, Na, K; SHE = standard hydrogen electrode. ^2^ Values obtained in ether-based electrolytes. ^3^ S. Am. = South America. ^4^ Analogously, the prices of sodium and potassium carbonate were also consulted at the link provided in ref. [33], replacing lithium by sodium or potassium. Data: November 2024, for Europe.

Nonetheless, KIBs also present some disadvantages compared with NIBs (as reflected in Table 1), such as (i) the low melting point of K metal, reducing the maximum operating temperature of the cell to 60 °C (in case the anode is K metal), (ii) a bigger ionic radius, requiring cathode and anode materials with larger cavities to diffuse the K^+^ and not hinder its kinetics, and (iii) the higher cost of K-based carbonate (typically used as a precursor in the synthesis of K-based materials), while it is still much lower than the Li one.

**Figure 1:**
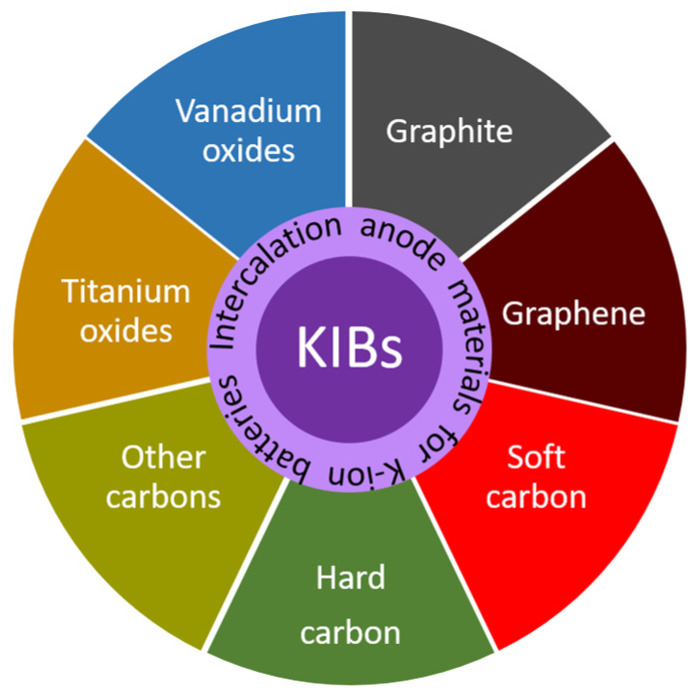
An overview of the intercalation anode materials under investigation for KIBs addressed in this review.

Although KIBs are mainly envisaged for large-scale applications, a recent techno-economic analysis based on Hurlbutt et al.’s model [34], carried out by Pasta et al. [19], also suggested that this technology could rival/compete with commercial LiFePO_4_/graphite LIBs, i.e., cobalt-free LIBs, for low-range EV applications, both in terms of cost and specific energy. In fact, efforts are being made in this regard, and, for example, the Group1 US company announced in 2024 its first production of 18650 KIBs, composed of a graphite anode and a Prussian White cathode [35]. However, KIBs still face several challenges that need to be overcome before entering the real market, such as limited energy and power density, low K-ion diffusion in solid electrodes, poor rate performance, large volume variations upon potassiation/depotassiation, and battery safety hazards [18]. With the common goal of KIBs reaching the same destination as LIBs, i.e., a real application, research, especially in electrode materials, has been hectic since 2015. So far, the main classes of cathodes under investigation are layered transition metal oxides, Prussian blue analogues, polyanionic compounds, and organic materials [36,37]. In this review, we will explore anode materials, focusing on the different types of intercalation materials (see Figure 1), which are the most promising to date from our point of view. Although intercalation anode materials have limited capacity compared with alloy and conversion materials, and hence, limited energy density, they exhibit much greater structural stability, which prolongs the cycle life. Herein we provide a compilation of an updated bibliography, highlighting the most relevant candidates based on the most recent advances. In addition, the shortcomings and key issues of these materials are also addressed along with possible strategies and future directions to follow. To the best of our knowledge, this is the first review reported on the intercalation anode materials for KIBs.

## 2. Carbon-Based Intercalation Anodes

### 2.1. Graphite

**Figure 2:**
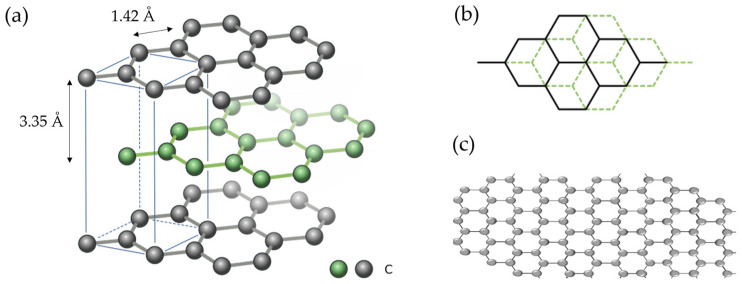
(**a**) AB stacked graphite structure, where the C-C distance (1.42 Å) in the hexagonal close packing within a graphene layer and the interplanar distance between graphene layers (3.35 Å) are observed. (**b**) Schematic top view of the AB layer stacking structure of graphite (figures adapted from Josef Sivek [40]). (**c**) Graphene monolayer.

Graphite is one of the allotropes in which carbon naturally crystallizes [38]. It is a 3D (three-dimensional) material with a layered structure formed by stacked graphene layers (as shown in Figure 2a,b) where alkali ions can be intercalated, forming GICs (graphite intercalation compounds) [39]. Additionally, this material benefits from electronic and thermal conductivity [38].

As mentioned, fortunately, potassium can be reversibly inserted into graphite. The formation of potassium–GICs (K-GICs), including stage I KC_8_, was reported back in the 1930s [41]. Nevertheless, the electrochemical intercalation of K^+^ in graphite at RT (room temperature) was not outlined until 2015 [31,32,42]. Using commercially available synthetic graphite as the active material of the working electrode, potassium metal as the counter/reference electrode, and 0.8 M KPF_6_ in EC:DEC 50: 50 as the electrolyte, Jian et al. achieved a depotassiation capacity of 273 mAh g^−1^ at C/40, which is close to the theoretically expected value (279 mAh g^−1^; see Table 2 for more details) [32]. It is worth noting that the theoretical capacity of graphite in KIBs (to form KC_8_) is lower than that of LIBs (to generate LiC_6_) [19], as reflected in Table 1, and that the voltage hysteresis increases as follows. Whereas K^+^ intercalation occurs at similar potentials (~0.2 V vs. K^+^/K) to the lithiation in LIBs (~0.1 V vs. Li^+^/Li), K^+^ extraction happens at ca. 0.3 V vs. K^+^/K (compared with 0.1 V vs. Li^+^/Li) [17]. Although the slightly higher intercalation potential in KIBs could be beneficial to avoid metal plating, a low ICE (initial Coulombic efficiency) of ca. 60%, fast capacity fading due to a large volume expansion, and a moderate rate capability were also observed.

#### 2.1.1. Mechanism

**Figure 3:**
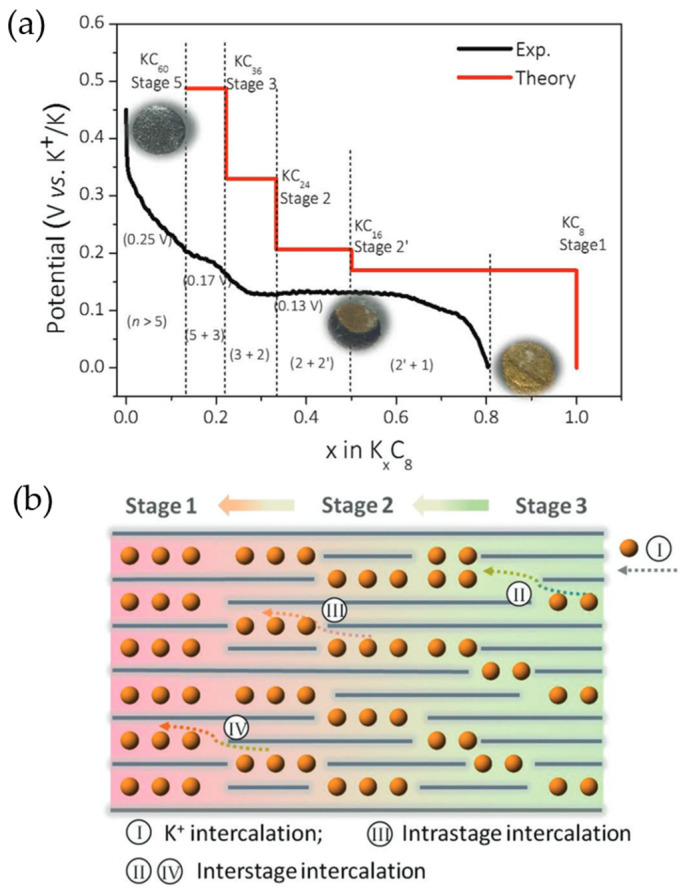
(**a**) Potential of the K-GICs vs. “x” in K_x_C_8_ (experimental results are plotted in black and DFT calculations in red). (**b**) Stage transition model for K-GICs. Step I: K^+^ intercalation, Step II: interstage intercalation (stage 3–stage 2), Step III: intra-stage intercalation (stage 2–stage 2′), and Step IV: interstage intercalation (stage 2–stage 1). Reproduced with permission from [45].

The K^+^ storage mechanism is still controversial. According to Jian’s ex situ XRD (X-ray diffraction) experiments, after the KC_36_ and KC_24_ intermediate phases, KC_8_ is formed at the end of the potassiation, and this transformation is reversibly reverted upon depotassiation (except for KC_24_, which was not detected) to obtain graphite less crystalline than the pristine one [32]. In fact, graphite potassiation could be described as C_graphite_ → KC_36_ (stage III) → KC_24_ (stage II) → KC_8_ (stage I) [32], in agreement with the K-intercalated graphite stages reported previously for vapor pressure experiments [43]. Another mechanism, proposed by Luo et al. based on a combination of ab initio DFT (density functional theory) calculations and electrochemical characterization, also divided the storage of K^+^ into three possible steps: C_graphite_ → KC_24_ (stage III) → KC_16_ (stage II) → KC_8_ (stage I) [42]. However, Zhao considered the intercalation of K^+^ in graphite as a four-step process: C_graphite_ → KC_48_ → KC_36_ → KC_24_ → KC_8_ [23,44]. Apparently, the most plausible theory was reported by Liu et al., who detected the coexistence of multiple stages by in situ XRD and Raman spectroscopy and proposed—on the basis of DFT calculations—an intrastate transition of stage II K-GIC (as illustrated in Figure 3): C_graphite_ → KC_36_ (stage III) → KC_24_ (stage II) → KC_24_/KC_16_ (stage II) → KC_8_ (stage I) [45].

In any case, there is a consensus that the most stable stoichiometry and fully potassiated species formed is KC_8_. Contrasting with LIBs, where LiC_6_ has the highest lithiation extent, KC_6_ would never be reached electrochemically within the voltage window typically used. Indeed, if more negative potentials were applied, K metal plating would occur instead [42]. At full K^+^ intercalation, graphite experiences a volume expansion about six times higher (ca. 60%) than that of Li^+^ intercalation (11%) [44], resulting in enlarged interlayer distances of 5.35 Å for KC_8_ compared with the 3.5 Å for LiC_6_ [16,46].

#### 2.1.2. Diffusion Coefficients, Formation Enthalpy, and Safety

Interestingly, DFT calculations have revealed that KC_8_ has a higher diffusion coefficient (2 × 10^−10^ m^2^ s^−1^) and lower formation enthalpy (−27.5 kJ mol^−1^) compared with LiC_6_ (1.5 × 10^−15^ m^2^ s^−1^ and −16.4 kJ mol^−1^) [46,47], suggesting easier intercalation of K^+^ in graphite and a superior rate performance of KIBs regarding LIBs. For the sake of comparison, the calculated enthalpies of formation of NaC_6_ and NaC_8_ are positive (respectively, +20.8 and +19.9 kJ mol^−1^), pointing out the instability of these phases [47], which is in accordance with the experimental results, since Na^+^ does not easily intercalate into graphite nor does it exhibit a high capacity as Li^+^ and K^+^ do [48,49]. The diffusion coefficients, however, have intermediate values (2.8⋅10^−12^ and 7.8⋅10^−13^ m^2^ s^−1^, respectively, for NaC_6_ and NaC_8_) between those for LiC_6_ and KC_8_ [46].

More importantly, analogous to LIBs, K metal plating may occur in the anode of KIBs in an overcharged state. Since K metal is known to react violently (more than Li and Na), safety is an inherent concern when considering the practical use of KIBs. Nonetheless, the greater difference between the potential at which K^+^ intercalates into graphite and the potential at which metallic K plating occurs could reduce the risk of dendrite formation in KIBs compared with LIBs. Furthermore, in the event that K plating happened, the high reactivity of the K metal would favor its quick reaction with the electrolyte, leading to the disappearance of most of the plated metal before causing a short circuit. Or, if a short-circuit were to occur, K metal could act as a fuse, melting—due to its low melting point (63.4 °C, Table 1)—and thus stopping the short circuit before serious thermal runaway took place [19,25,44]. Anyway, to verify these hypotheses, safety studies in KIBs are urgently required. In the meantime, there are still reports that prudently consider the high reactivity of K metal a safety hazard [18,50]. Of the few studies published to date in this regard, the onset of thermal runaway for graphite in KIBs has been reported to happen at lower temperatures than the graphite anode of commercial LIBs (100 °C vs. 50–450 °C), although generating significantly less heat (395 J g^−1^ vs. 1048 J g^−1^) [51]. From our viewpoint, a possible attempt to prevent or avoid thermal runaway could be, for instance, to replace the liquid electrolyte with a non-flammable and/or fire-retardant liquid electrolyte or switching toward solid-state electrolytes, which are intrinsically safer.

**Figure 4:**
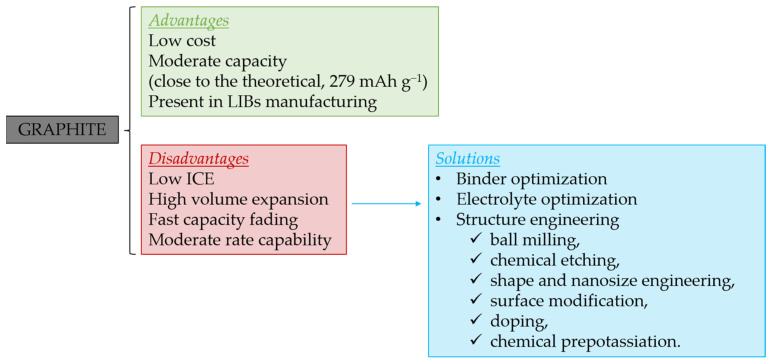
Advantages and disadvantages of graphite in KIBs and possible solutions to the latter.

Figure 4 briefly summarizes the main advantages and disadvantages of graphite in KIBs as well as the possible solutions applied to overcome its shortcomings, which will be discussed in more detail in the subsequent sections.

#### 2.1.3. Binder and Electrolyte Optimization to Extend the ICE and Cyclability

**Figure 5:**
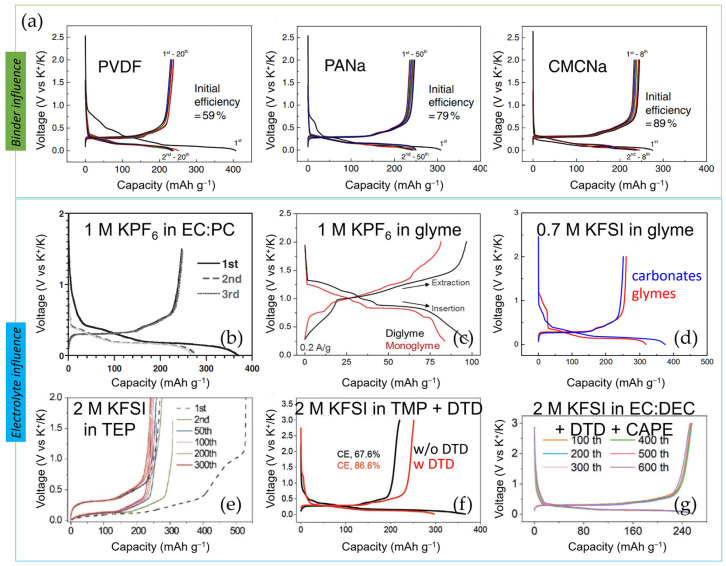
Influence of the (**a**) binder: PVDF, PANa, and CMCNa (figures adapted from Shinichi Komaba [31]), and electrolyte: (**b**) 1 M KPF_6_ in EC:PC (adapted from Jin Zhao [44]), (**c**) 1 M KPF_6_ in glyme (figure adapted from Adam P. Cohn [54]), (**d**) 0.7 M KFSI in glyme (adapted from Tomooki Hosaka [59]), (**e**) 2 M KFSI in TEP (figure adapted from Sailin Liu [64]), (**f**) 2 M KFSI in TMP and 6 wt.% DTD (adapted from Gang Liu [70]), and (**g**) 2 M KFSI in EC: DEC with 2 wt.% DTD and 0.5 wt.% CAPE (figure adapted from Mingyuan Gu [71]) on the voltage profile of graphite.

Optimization of *binders* and electrolytes can effectively upgrade the ICE, cyclability, and rate capabilities of graphite (see Table 2). In this sense, Komaba’s group illustrated that, despite the almost identical capacity and redox potentials observed, the substitution of the PVDF (polyvinylidene fluoride) binder with PANa (sodium polyacrylate) or CMC (sodium carboxymethylcellulose) had a preformed SEI (solid electrolyte interphases) effect, leading to an increase in ICE (from 59% to 79 or 89%, respectively), as well as enhanced cycle stability (>200 mA h g^−1^ for 50 cycles) and rate capability with PANa (see Figure 5a) [31].

*Electrolytes* also have a tremendous impact on the formation of a stable SEI in terms of composition, morphology, and ionic conductivity, thereby affecting the long-cycle performance of the cell [52]. Along with the most-used K salt, KPF_6_, which prevents the corrosion of the Al current collector [19], different carbonate electrolyte mixtures (EC:DMC, EC:DEC, and EC:PC 1:1 *v/v*, where DMC = dimethyl carbonate) have been tested as solvents, obtaining the highest ICE (66.5%) (Figure 5b) and best capacity retention (220 mAh g^−1^ after 200 cycles) in EC:PC, among the three tested [44]. Significant decomposition of DEC, occurring both at the Kǁelectrolyte and the graphiteǁelectrolyte interphases, has been confirmed by ^1^H-NMR (proton nuclear magnetic resonance) [53]. By limiting the lower voltage cutoff to 0.25 V vs. K^+^/K and moving to ether-based electrolytes (see Figure 5c) such as diglyme (diethylene glycol dimethyl ether, also known as DEGDME) or DME (1,2-dimethoxyethane, also referred to as glyme), excellent capacity retention of 95% over 1000 cycles and up to 100 mAh g^−1^ of capacity was achieved at 2 A g^−1^ in free-standing multi-layered graphene foam electrodes [54]. Although K^+^ is intercalated into graphite at a higher operating voltage in the ether-based DME than in the carbonate-based EC:DMC electrolyte (~0.7 V vs. ~0.2 V), and the specific capacity is reduced because ether-based electrolytes co-intercalate with K^+^ in graphite, the diffusion coefficient of K^+^ is greater (3 × 10^−8^ cm^2^ s^−1^ vs. 6.1 × 10^−10^ cm^2^ s^−1^), the volume variation is smaller (7.7% in DME vs. 63% in EC:DMC), and an almost negligible SEI is apparently formed (even after 350 cycles), as indicated by its low SEI resistance (< 10 Ω) [55]. These results are consistent with a thin SEI detected by XPS (X-ray photoelectron spectroscopy) and TEM (transmission electron microscopy) on the surface of the graphite in DEGDME electrolytes [56]. Enhanced K^+^ storage in graphite has also been reported by the introduction of cyclic ether, such as THF (tetrahydrofuran), as cosolvent [57].

Despite the tendency of KFSI-based electrolytes at conventional concentration (~1 M) to cause Al current collector corrosion at potentials > 4 V vs. K^+^/K, KFSI (potassium bis(fluorosulfonyl)imide) is the second most used salt in KIBs [52]. The work by Wu and coworkers, who first proposed the use of a high-concentration KFSI in the DME electrolyte for the K metal anode and found that this combination allowed reversible platting and stripping on it without dendrite formation during ca. 200 cycles, encouraged further studies [58]. Precisely, in highly concentrated KFSI in DME (Figure 5d), Komaba et al. recently reported the exceptional performance of graphite, exhibiting 260 mAh g^−1^ stable for 300 cycles (i.e., 99.9% capacity retention) and an impressive rate capability (200 mAh g^−1^ at 5C) [59]. The reasons for such good results are the following: (i) Highly concentrated electrolytes have high oxidation resistance, and thus, higher voltage window stability. (ii) In addition, in highly concentrated KFSI-based electrolytes, as a result of the decreased activity of the solvent, the Al^3+^ dissolution (causing Al corrosion) is suppressed, and the DME co-intercalation is prevented [59]. Pasta’s group also proved superior performance using KFSI in Pyr_13_FSI (N-butyl-N-methylpyrrolidinium bis(fluorosulfonyl)imide) IL (ionic liquid) electrolyte. They observed a reversible capacity of 246 mAh g^−1^, maintained over 400 cycles (99% capacity retention), and a stable average CE of 99.94% [60]. Even more incredible, in the high-concentration electrolyte KFSI in EMC (ethylene methylene carbonate), at a molar ratio of 1:2.5, graphite is capable of displaying a high reversible capacity (ca. 255 mAh g^−1^) and outstanding cyclability in KIBs, with negligible capacity decay during 2000 cycles (equivalent to ca. 17 months), most likely due to the formation of a more stable and robust inorganic-rich SEI [61]. In general, KFSI-based electrolytes offer higher conductivity than the KPF_6_ analogues in various solvents, including PC, DME, or EC:DEC [59], and a thinner, more homogeneous, and stable SEI [61,62]. Conversely, KPF_6_-derived SEI is rich in unstable organic alkyl carbonates [62]. In this context, aware of the decisive role of KFSI salt on SEI formation but its costly price compared with KPF_6_, Komaba et al. tested KPF_6_/KFSI binary-salt electrolytes in carbonaceous ester solvents. In mixtures with KPF_6_/KFSI ratios ≥ 3, particularly 0.75 m K(PF_6_)_0.9_(FSI)_0.1_ and 1 m K(PF_6_)_0.75_(FSI)_0.25_ in EC:DEC, better ICE (87% and 89%, respectively) and rate performances were observed for graphite negative electrodes. Furthermore, the resulting negligible Al corrosion at high voltage enabled the reversibility of a graphiteǁK(PF_6_)_0.75_(FSA)_0.25_/EC/PCǁK_2_Mn[Fe(CN)_6_] full cell for 500 cycles [63]. Recently, Guo’s group succeeded in using TEP (triethyl phosphate), a non-flammable solvent, with a moderate concentration (2 M) of KFSI salt for graphite anodes in KIBs (see Figure 5e), achieving near-theoretical capacity at 0.2 C and 90% retention after 300 cycles [64]. They showed, as well, unprecedented stability of a graphite anode by using a moderately concentrated KFSI in TMP (TMP = trimethyl phosphate) in an 8: 3 molar ratio fire-retardant and non-flammable electrolyte, which enabled the retention of 74% of the initial capacity over 24 months of cycling (ca. 2000 cycles) at 0.2 C [65]. According to the authors, this work represents the longest calendar life ever reported for graphite in KIBs and somehow reinforces the practicability of KIBs. Another remarkable work is the research by Lu et al., who prepared commercial graphite with an *artificial inorganic SEI* by simply keeping the graphite anode in contact with a K metal foil and soaking it in 3 M KFSI in DME for 15 h. This surface-modified graphite exhibited an ICE of 93% and almost 1000 cycles with little capacity decay when tested in a half-cell configuration and using an industrial carbonate-based electrolyte (0.8 M KPF_6_ in EC:EMC, 1:1 *v/v*) [66].

Regarding *additives*, FEC (fluoroethylene carbonate), which is well known for facilitating the formation of a stable SEI in both LIBs and NIBs [67], unexpectedly reduces the reversible capacity and increases the capacity degradation in graphite KIB half cells, possibly due to the formation of a highly resistive passivating layer [68]. On the contrary, the incorporation of 1 or 10 wt.% of KFSI or DMSF (dimethyl sulfamoyl fluoride) to conventional 0.75 m KPF_6_ in EC:DEC electrolyte has shown improved CE and discharge capacities in graphite performance. In a full cell configuration, graphite ǁK(PF_6_)_0.75_(FSI)_0.25_/EC/DECǁK_2_Mn[Fe(CN)_6_], the presence of 10 wt.% DMSF led to 82.4% capacity retention and high CE [69]. On the other hand, the addition of 6 wt.% DTD (ethylene sulfate) made the utilization of non-concentrated 1 M KFSI in a non-flammable and fire-retardant TMP solvent compatible with a graphite anode (Figure 5f), which extended its cyclability, allowing it to show 272 mAh g^−1^ at 0.2C with almost negligible decay for 100 cycles. According to the authors’ findings, this positive effect of DTD was not due to the formation of a stable SEI but rather to the suppression of the co-insertion of K^+^-TMP into graphite [70]. In the previous works highlighted here, the additive is reduced first, forming the SEI and minimizing the reduction reactions of the solvent(s) composing the electrolyte. However, this year, Gu et al. have reported a new concept of additives [71]. The proposed alternative approach consists of adding caffeic acid phenethyl ester (CAPE) to DTD (Figure 5g), which, under reduction (in contact with electrons), polymerizes on the graphite surface, forming a homogeneous and flexible SEI, thus enabling the SEI to adapt to the possible volume change upon cycling and, in turn, providing greater structural stability to the graphite, as indicated by the stable capacity obtained over 1500 cycles at 100 mA g^−1^. In 2024, an electrolyte based on a solvent with a weak solvation effect, such as 1,2-diethoxyethane (DEE), has also been proposed, in combination with a 1,1,2,2-tetrafluoroethylene-2,2,3,3-tetrafluoropropyl ether (TTE) additive [72]. As the desolvation energy is reduced, the ionic conductivity of the electrolyte and the SEI formed increases, even at low temperatures. Due to the TTE additive, in addition to forming a uniform SEI, the graphite exhibited a specific capacity of 230 mAh g^−1^ at −5 °C (85.8% capacity retention with respect to the capacity at room temperature) and 273 mAh g^−1^ at 45 °C. The excellent electrochemical performance of graphite over a wider temperature range with this new concept of electrolyte chemistry clearly indicates the need to develop advanced electrolytes beyond carbonate-based ones.

In summary, it is evident that the electrolyte solvents and additives accompanying the selected salt, as well as the binder, play a crucial role in the electrochemical properties. Therefore, the adequate choice of the binder (to prepare the graphite anode electrodes) and the electrolyte salt (and adjustment of its concentration), together with the use of a suitable solvent or co-solvents and additives, optimizing in any case their proportion, can considerably improve the SEI composition, thereby improving the ICE and extending the cycle life of the battery. Although the attention in this review has been focused on organic electrolytes, ILs and solid-state electrolytes represent an alternative—the latter, especially for K metal batteries—on which further information can be found in other comprehensive reviews exclusively dedicated to the electrolyte [73,74,75].

#### 2.1.4. Graphite Structure Engineering to Enhance Its Performance

We have just seen that, aiming at a more stable SEI, mainstream research has mainly focused on the electrolyte. Although less common, some efforts have also been devoted directly to transforming or modifying the structure of the graphite. Several approaches, including mechanical methods (ball milling), chemical transformation (chemical etching and oxidation), shape and nanosize engineering, surface modification, doping, and chemical pre-potassiation, are among the engineering strategies utilized (see Table 2) to remarkably upgrade graphite’s cyclability and C-rate performance as an anode in KIBs.

*Ball milling* (BM) can improve, to some extent, the performance of graphite for K^+^ storage. As a result of the mechanical exfoliation of graphite achieved by this method, Carboni et al. showed an enhanced capacity and capacity retention compared with electrode materials mixing with a manual agate mortar [76]. However, probably due to the minimal structural modification in the graphite and because the electrodes were composed of only 90 wt.% graphite and 10 wt.% PVDF binder, the capacity retention fell below 80% in less than 100 cycles. Shortly after, an acetone-based low-energy wet BM method, developed by Rahman and coworkers, led to obtaining thin graphite flakes with a high surface area capable of delivering 227 mAh g^−1^ after 500 cycles (98% capacity retention) at 100 mA g^−1^ and with outstanding performance at high rates (226 mAh g^−1^ at 4 A g^−1^) in 0.8 M KPF_6_ in EC:DEC 1:1 *v/v* vs. K [77].

Another strategy to change the structure of graphite is *to expand its c-axis*. In this context, expanded graphite has been synthesized by *chemical etching* after mixing high-purity graphite with KOH and posterior high-temperature (850 °C) treatment under an Ar atmosphere. After this chemical activation, the particle size decreased from micrometers to nanometers, the *c*-axis interplanar distance enlarged to 0.358 nm, and the number of pores increased, resulting in a ca. 7 times improved K^+^ diffusion coefficient [78]. Mildly expanded graphite (MEG-x) with adjustable interlayer spacing, obtained by Kang’s group via wet chemical oxidation with permanganate and subsequent annealing at 650 °C, has also proven remarkable for K^+^ storage. In particular, MEG-2 prepared using a permanganate/graphite ratio of 2: 3 displayed good specific capacity (226 mAh g^−1^) and capacity retention (85% and 72%, respectively, after 100 and 200 cycles) at 100 mA g^−1^ [79]. In a more recent study, commercially available expanded graphite with enlarged interlayer distances of 0.387 nm (compared with the 0.34 nm of regular graphite) provided almost 180 mAh g^−1^ for 500 cycles at 200 mA g^−1^ in 1 M KFSI in EC:DEC [80]. Through strong initial oxidation of graphite and posterior pyrolysis at several temperatures, Xing’s group has also prepared expanded graphite. A good C-rate performance, with reversible capacities of 303 mAh g^−1^ at 10 mA g^−1^ and 105 mAh g^−1^ at 1 A g^−1^, was observed for the expanded graphite pyrolyzed at 750 °C, which presented a *d*-spacing of 0.37 nm. The adsorption process occurred mainly above 0.3 V, whereas intercalation mostly happened below this potential. In addition, 160 mAh g^−1^ could be maintained after 1000 cycles at 200 mA g^−1^, with a capacity decay of only 0.02% per cycle [81].

*Shape and nanosize engineering* is another methodology to follow. In this sense, using chemical vapor deposition, Xing et al. synthesized a unique polynanocrystalline graphite with highly graphitic nanodomains along the *c*-axis, randomly packed to form micron-sized particles, which exhibited poorer C-rate capability but better capacity retention (50% vs. 6% after 300 cycles) than regular graphite [82]. Interestingly, Guo’s group designed a flexible, ultralight, current-collector-free, and binder-free graphite anode by simply drawing on filter paper with a pencil. As the inert weight of the electrode was reduced, a capacity improvement close to 200% was observed over that of electrodes containing current collectors. With this innovative anode, a high reversible capacity of 230 mAh g^−1^ at 0.2 A g^−1^ was achieved, along with fairly good capacity retention (75% over 300 cycles at 0.4 A g^−1^) and excellent rate performance (66% capacity retention at 0.5 A g^−1^) [83].

*Surface modification*, such as coating the graphite surface with Al_2_O_3_ by ALD (atomic layer deposition), allows building a stable SEI and enhances the K^+^ storage performance of the graphite anode [84].

*Doping* is another well-known option to transform the electrochemical properties of graphite. For instance, the incorporation of N heteroatoms creates structural defects and/or doping sites in the carbon lattice, contributing to the absorption of K^+^ [85] and thus boosting its capacity. The effect of different concentrations and configurations of N-doping was studied in self-supported graphite foam. The enlarged interlayer spacing (~3.46 nm) via N-doping and the holey structures induced by pyridinic and pyrrolic nitrogen contributed to improved K^+^ storage, offering reversible capacities of 248 mAh g^−1^ at 10 mA g^−1^, notable cycling stability (86% capacity retention over 200 cycles at 40 mA g^−1^), and also superior rate capability (fast K^+^ diffusion in the electrode) compared with graphite [86]. Li et al. prepared N-doped graphitic carbons at different carbonization temperatures (referred to as ENGC-T). In particular, ENGC-850, with an expanded interlayer distance of 0.358 nm, an ultrahigh edge/N ratio (76.6%), and N-5 (or pyrrolic N) content (ca. 42%), exhibited prolonged cycle life, delivering 189 mAh g^−1^ at 2C after 2200 cycles. Conversely, a not so good ICE of 74% (at 0.5 A g^−1^) was observed, even though pre-potassiation [66] was applied. It is also important to note that the process that occurred was mainly capacitively controlled [87].

*Chemical pre-potassiation* has also been reported. In a recent study, potassium-enriched graphite (KRG), prepared electrochemically by over-discharging it to a certain capacity (250 mAh g^−1^), was able to surpass the capacity of the original graphite, reaching ~520 mAh g^−1^ in 5 m KFSI in EC:DEC. Nonetheless, no more than 50 cycles were shown. A full cell was constructed by pairing the enriched graphite with a Prussian blue cathode, which could deliver 108 mAh g^−1^ after 180 cycles at 100 mA g^−1^ [88].

Table 2 summarizes the most relevant works covered in this review related to the use of graphite as an anode in KIBs, including strategies to improve its electrochemical performance, both through electrolyte/binder optimization and structure modification.

Graphite has proven to be a good candidate as an anode material in KIBs. Nonetheless, before reaching commercialization, certain aspects must be addressed, such as improving its long-term cyclability and C-rate performance, eliminating any possible risk of K plating, and gaining a deeper understanding of its reaction mechanism and of the structure and composition of its SEI, the latter being closely related to the electrolyte (whether liquid or solid) and the binder selected, which are not yet optimized.

**Table 2:** Electrochemical performance of graphite anode materials in KIBs. N/A = not available.

				Cyclability		Rate Capability	
Anode Material	Electrolyte	Binder	ICE	Capacity (mAh g^−1^)	Cycle Number@ Current Density (A g^−1^)	Capacity (mAh g^−1^)@ Current Density (A g^−1^)	Ref.
Graphite (TIMCAL)	0.8 M KPF_6_/EC:DEC	PVDF	57.4%	~100	50 cycles@0.14	~75@0.28	[32]
Graphite (GT)	0.5 M KPF_6_/EC:DEC	PVDF	74%	207	N/A	~88@0.2	[42]
Natural graphite	1 M KFSI/EC:DEC	PANa	79%	~230	50 cycles@0.028	225@4.19	[31]
Natural graphite	1 M KFSI/EC:DEC	CMC	89%	~230	8 cycles@0.028	N/A	[31]
Natural graphite	1 M KFSI/EC:DEC	PVDF	59%	~230	20 cycles@0.028	N/A	[31]
Graphite	1 M KPF_6_/EC:PC	Na-alginate	66.5%	~230	200 cycles@0.02	N/A	[44]
MLGF ^1^	1 M KPF_6_/DEGDME	Free	73%	95	1000 cycles@2	~80@10	[54]
Natural graphite	1 M KPF_6_/DME	CMC	87.4%	73	3500 cycles@2.8	87@2.8	[55]
Flake graphite	0.5 M KPF_6_/DEGDME	PVDF	90%	80.8	50 cycles@0.025	N/A	[56]
Graphite	0.8 M KPF_6_/EC:DEC:THF	PVDF	54%	196	100 cycles@0.093	~80@0.28	[57]
Graphite	7 m KFSI/DME	PANa	~78%	~260	300 cycles@0.025	~200@0.75	[59]
Graphite	1 m KFSI/Pyr_1,3_FSI	PAA ^2^	~80%	233	400 cycles@C/5	~216@2C	[60]
Graphite	2.5 M KFSI/EMC	CMC	~80%	255	2000 cycles@0.093	N/A	[61]
Graphite	1 m K(PF_6_)_0.75_(FSI)_0.25_/EC:DEC	PANa	89%	270	100 cycles@0.025	N/A	[63]
Graphite	2 M KFSI/TEP	PVDF	88.5%	~250	300 cycles@0.056	~130@0.56	[64]
Graphite	KFSI/TMP (8: 3)	PVDF	~58%	~204	2000 cycles@0.056	~100@0.56	[65]
Graphite	0.8 M KPF_6_/EC: EMC + artif. SEI	CMC	93%	~260	1000 cycles@0.1	~100@0.5	[66]
Graphite	0.75 m KPF_6_/EC:DEC + 10 wt.% DMSF	CMC	~84%	~240	25 cycles@0.1C	~225@1C	[69]
Graphite	0.75 m KPF_6_/EC:DEC + 10 wt.% KFSI	CMC	~89%	~240	25 cycles@0.1C	~225@1C	[69]
Graphite	1 M KFSI/TMP + 6 wt% DTD	CMC+SBR	86.6%	272	100 cycles@0.028	~225@0.67	[70]
Graphite	1 M KFSI/EC: DEC + DTD + CAPE	CMC	~76%	~250	1500 cycles@0.1	N/A	[71]
Graphite	1 M KPF_6_/DEE + 1 wt.% TEE	N/A	~75%	~250	220 cycles@0.1	N/A	[72]
BM graphite	0.8 M KPF_6_/EC:DEC	PVDF	61%	150	100 cycles@0.025	~200@0.25	[76]
BM graphite flakes	0.75 M KPF_6_/EC:DEC	CMC	74%	222	500 cycles@0.1	226@4	[77]
Activated carbon	0.8 M KPF_6_/EC:DEC	PVDF	~78%	100	100 cycles@0.2	114@0.4	[78]
Expanded graphite	0.5 M KPF_6_/EC:DEC	PVDF	~51%	192	100 cycles@0.1	88@1.5	[79]
Expanded graphite	1 M KFSI/EC:DEC	CMC	81.6%	228	200 cycles@0.05	180@0.2	[80]
Expanded graphite	0.8 M KPF_6_/EC:DEC	PVDF	39.5%	158	1000 cycles@0.2	106@1	[81]
Polynanographite	0.8 M KPF_6_/EC:DEC	CMC	54.1%	75	240 cycles@0.1	43.2@0.5	[82]
Pencil-trace anode	0.8 M KPF_6_/EC:DEC	Free	~65%	~170	350 cycles@0.4	~115@1	[83]
N-doped GT foam	1 M KFSI/EC:DEC	Free	59%	~170	200 cycles@0.4	112@0.2	[86]
Rich N-doped GT	1 M KFSI/DME	PVDF	48.7%	266	100 cycles@0.5	112 @0.2	[87]
K-enriched GT	5 m KFSI/EC:DEC	PVDF	~85%	~215	200 cycles@0.025	N/A	[88]

^1^ MLGF = Multilayered graphene foam. ^2^ PAA = Polyacrylic acid.

### 2.2. Graphene

Graphene is a 2D (two-dimensional) carbon material with sp^2^ hybridization (see Figure 2c), which displays ultrahigh surface-to-mass area (~2600 m^2^ g^−1^), high electronic conductivity, excellent mechanical properties, and the potential to store K ions. In theory, its capacity to store K^+^ is higher than that of graphite, since potassium absorption can occur on both sides of the graphene [23,89]. Therefore, graphene is an appealing anode material currently under investigation for KIBs.

*Reduced graphene oxide (rGO).* Advantageously, rGO presents a larger interlayer distance compared with graphite. Electrochemical intercalation of K^+^ into a graphene-derived material, rGO, was reported for the first time in 2015. Although this free-standing rGO film, with an interlayer distance of 3.66 Å, showed a first-cycle reversible capacity of 222 mAh g^−1^ at 5 mA g^−1^, its cyclability was mediocre, and its rate capability was poor [42]. To improve this low K^+^ ionic conductivity exhibited by rGO, Simon et al. designed and synthesized a 3D rGO aerogel using a freeze-drying method. Apparently, because it avoided restacking (a common problem of graphene), the 3D rGO displayed an initial capacity of 267 mAh g^−1^ at C/3 (26 mA g^−1^) and an improved cycle life, retaining ca. 80% of this capacity after 100 cycles. Even at a higher C-rate of 125 mA g^−1^, 125 mAh g^−1^ was obtained after 500 cycles (see further details in Table 3) [90]. Interestingly, the electrochemical performance of rGO in KIBs is correlated with its microstructure, which turns out to be temperature-dependent. As a result of its expanded interlayer distance and graphite-like structure, rGO graphitized at 2500 °C (rGO-2500) was able to exhibit an ultralong cycle life of 2500 cycles [91].

*Few-layer graphene (FLG).* Electrochemical K^+^ storage has also been evaluated in FLG grown by CVD (chemical vapor deposition) on Ni foam [92]. Although this binder-free material delivered a reasonable capacity of 210 mAh g^−1^ at 0.1 mA g^−1^, only 140 mAh g^−1^ was retained after an elapsed 100 cycles, and its rate testing was also not good. Nonetheless, it allowed the researchers to elucidate the K^+^ insertion mechanism into the FLG with the help of in situ Raman, which supported Jian’s proposal of the K^+^ storage mechanism [32] for graphite. Superior cycling stability was achieved, however, with 3D FLG microspheres (FLGM) prepared from tetraphenyltin using a sulfur-assisted methodology. Of the initial 285 mAh g^−1^ exhibited at 50 mA g^−1^, 255 mAh g^−1^ (89%) was still maintained over 100 cycles, and at higher current densities, such as 200 mA g^−1^, negligible capacity loss was observed [93].

*Doping.* Fortunately, the electrochemical performance of graphene can be tailored through heteroatom functionalization [94]. As we discussed in the graphite section, heteroatom doping is an efficient strategy to increase the active surface area and boost the capacity and reaction kinetics in carbon-based materials [23]. F-doped [94], N-doped [95,96], S-doped [97], and even dual P/O-co-doped [98], N/P-co-doped [99,100], N/O-co-doped [101], and N/S-co-doped [102] graphene derivatives have been explored for the storage of K ions. Their ameliorated electrochemical properties are collected in Table 3, along with those of other graphene-based materials.

Even though heteroatom doping increases the chemical adsorption and conductivity of K^+^ in graphene-based materials, enhancing their electrochemical properties in KIBs, some of the still outstanding challenges associated with this type of materials are the lack of a defined voltage plateau (or the slope-shaped potential profile they present), a perceptible voltage hysteresis, and a low ICE due to an excessive electrolyte consumption.

**Figure 6:**
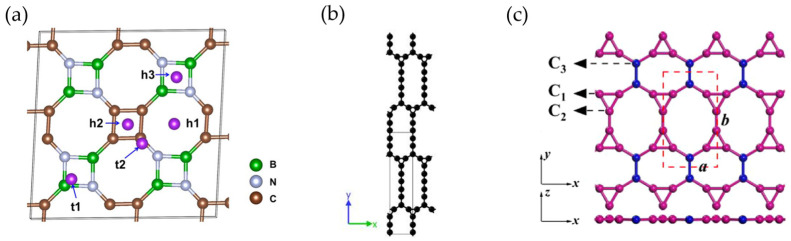
Structures of (**a**) T-graphene-like BC_2_N (reproduced from ref. [104]), (**b**) twin graphene (reproduced from ref. [107]), and (**c**) 2D TOD-graphene (reproduced with permission from ref. [106]). In T-graphene-like BC_2_N, purple balls represent the possible absorption sites of K. In TOD-graphene, top (upper image) and side views (bottom image) are shown, where blue and pink balls depict atoms from the graphene lattice and the kagome lattice, respectively.

Graphene is unlikely to be used alone as an anode material, although it is commonly found forming composites, in which it acts as a conductive carbon network. However, recent theoretical calculations have predicted the suitability of certain graphene-derived materials, such as boron-doped graphene [103], T-graphene-like BC_2_N monolayer [104], twin graphene [105], or TOD-graphene [106], as potential candidate anodes for KIBs. Among the boron-doped graphene species, B_5_C_27_ was found to have the highest work function and to possess a high theoretical specific capacity of 1131 mAh g^−1^. For its part, T-graphene is a carbon monolayer incorporating both C8 and C4 rings in its structure, which, through the incorporation of N and B atoms, can lead to the formation of a B-C-N nanosheet, i.e., forming a T-graphene-like BC_2_N monolayer (Figure 6a) capable of delivering 2196 mAh g^−1^. Twin graphene (Figure 6b) could be assimilated to bilayer graphene but with an additional level of complexity arising from the organic linkage of the two layers. According to DFT calculations, twin graphene could provide a theoretical capacity of 495.84 mAh/g and offer a low diffusion barrier of 0.290 V for K diffusion. On the other hand, TOD-graphene is a 2D material that integrates the kagome topology into the honeycomb lattice of graphene (Figure 6c) with remarkable potential K storage properties such as ultrahigh theoretical capacity (1115.8 mA h g^−1^) and a low diffusion barrier of 0.36 eV.

**Table 3:** Electrochemical performance of graphene-derived anode materials in KIBs.

					Cyclability		Rate Capability	
Anode Material	Electrolyte	Binder	ICE	Initial Capacity (mAh g^−1^)@ Current Density (A g^−1^)	Capacity (mAh g^−1^)	Cycle Number@ Current Density (A g^−1^)	Capacity (mAh g^−1^)@ Current Density (A g^−1^)	Ref.
S-s. rGO ^1^	0.5 M KPF_6_/EC:DEC	Free	~50%	~222@0.010	~107	150 cycles@0.010	50@0.1	[42]
S.s. rGO aerogel ^1^	0.7 M KPF_6_/EC:DEC	Free	44%	267@0.026	230	100 cycles@0.026	~100@0.523	[90]
rGO-2500	0.5 M KPF_6_/DEGDME	PVDF	62%	125@0.1	88.4	2500 cycles@0.1	~60@1.1	[91]
FLG ^2^ on Ni foam	0.8 M KPF_6_/EC:DEC	Free	N/A	~210@0.1	~140–150	100 cycles@0.1	~62@0.1	[92]
FLG microspheres	0.8 M KPF_6_/EC:DMC + artif. SEI	CMC	94%	285@0.05	230	1000 cycles@0.2	95@1	[93]
F-doped FLG foam	0.8 M KPF_6_/EC:DEC	PVDF	41.2%	356@0.05	166	200 cycles@0.5	213@0.5	[94]
N-doped FLG	0.8 M KPF_6_/EC:DEC	Free	~87%	270@0.05	210	100 cycles@0.1	~50@0.2	[95]
N-doped monolith	0.8 M KPF_6_/EC:DEC	CMC	~15%	487@0.02	150	1000 cycles@0.5	~180@5	[96]
S-s. S-doped rGO	1 M KPF_6_/EC:PC	Free	65%	456@0.05	361	50 cycles@0.05	224@1	[97]
P/O-doped graphene	1 M KClO_4_/EC:DEC	PVDF	22.6%	566@0.05	~400	600 cycles@0.5	222@1	[98]
N/P-doped MLG ^3^	1 M KPF_6_/EC:DEC	PVDF	15%	387@0.05	242	500 cycles@0.5	194@1	[99]
N/P-doped G on CC ^4^	1 M KPF_6_/EC:DEC + 5wt%FEC	Free	53%	366@0.025	281	1000 cycles@0.025	186@1	[100]
N/O-doped G-l CNC ^5^	1 M KPF_6_/DEGDME	CMC	N/A	~185@0.5	130	300 cycles@0.5	114@1	[101]
N/S-doped G nrbs ^6^	0.8 M KPF_6_/EC:DEC	PAA	55%	~267@0.5	224	500 cycles@0.5	212@1	[102]

^1^ S-s. = Self-standing. ^2^ FLG = Few-layer graphene. ^3^ MLG = Multi-layer graphene. ^4^ G = graphene, CC = carbon-cloth. ^5^ G-l = graphene-like, CNC = carbon nanocages. ^6^ nrbs = nanoribbons.

### 2.3. Soft and Hard Carbons

**Figure 7:**
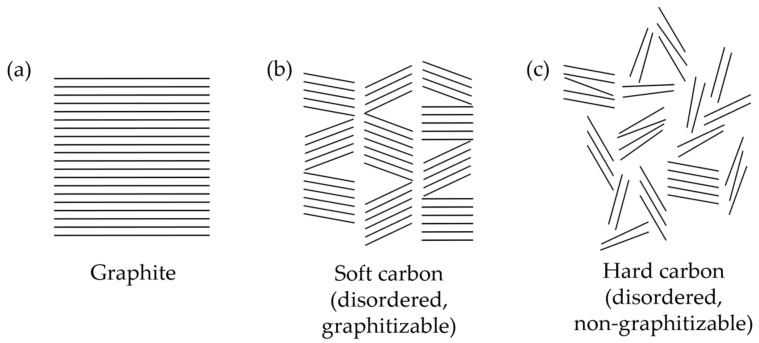
Schematic illustrations of the structure of (**a**) graphite, (**b**) soft (disordered, graphitizable) carbon, and (**c**) hard (disordered, non-graphitizable) carbon.

Soft and hard carbons differ primarily in their structure and ability to completely reconvert into graphite (Figure 7a) at temperatures above 2500 °C. Soft carbon (SC) is graphitizable, with long-range order (Figure 7b), high conductivity, and adjustable interplanar distance and crystallinity [19,108], whereas hard carbons (HC) are non-graphitizable materials with disordered structures (although short-range ordered) and large interlayer spacing (Figure 7c) [108,109]. Therefore, HC is characterized by displaying excellent cycle performance and SC by exhibiting superior rate performance.

*Soft carbon.* In addition to evidencing the reversible insertion of K^+^ into graphite, Ji’s group investigated the performance of SC in KIBs, which resulted in improved cycling stability and rate capability compared with graphite [32]. This SC, prepared by pyrolysis of PTCDA (3,4,9,10-perylene-tetracarboxylic acid-dianhydride) at 900 °C for 5 h, turned out to be less dense than graphite (1.6 g cm^−3^ vs. 2.3 g cm^−3^) and to present a favorable turbostratic structure with enlarged *d*-spacing (0.355 nm). Also using PCTA as the precursor, SC semi-hollow microrods were synthesized as anodes for KIBs at diverse sintering temperatures [110].

Pitch-derived SC, with high structural flexibility, was also identified as a promising high-performance anode material for KIBs. Its turbostratic lattice has a wide lattice spacing (3.49 Å) that favors rapid intercalation/deintercalation of K^+^ and prevents long-range lattice ordering—often observed in SC—thus conferring it greater structural and mechanical resilience compared with graphite. Consequently, longer cyclic stability was observed at 1C (ca. 0.28 A g^−1^) in contrast to graphite and HC [111]. In addition, a higher energy density compared with HC would be guaranteed if the voltage window were restricted below 1 V vs. K^+^/K, since, under these conditions, intercalation contributes significantly to the capacity, while surface chemical adsorption does not. Although a K^+^ intercalation mechanism similar to that of graphite was expected for SC, the authors found that different processes occurred, as the higher-order phases KC_24_ and KC_36_ were particularly difficult to form. In a recent study, the influence of the chemical structure of pitches was tackled, observing that the total absence or massive existence of aliphatic substituents in the pitch was conducive to bulk structures, while the existence of a certain amount of aliphatic substituents could instead lead to beneficial lamellar structures [112].

Recently, ultrathin 2D wrinkled soft carbon sheets (USCs), pitch-derived and synthesized using the template effect of melamine, have demonstrated high specific capacity and outstanding rate capability and reversibility. Despite its low ICE resulting from the formation of the SEI in the first cycle, its enlarged interlayer spacing facilitated the transport of K^+^, and the wrinkled structure prevented the stacking of 2D sheets, providing a buffer space for volume expansion [113]. In addition, PVC-derived SCs have also been produced to recycle this plastic waste (PVC = polyvinylchloride). Different carbonization temperatures (T = 600, 800, 1000, and 1200 °C) were used to prepare the resulting material, PVC-SC-T. The highest capacity retention was observed for the sample carbonized at a higher temperature, PVC-SC-1200, while the best rate capability was displayed by PVD-SC-800 [114]. In 2024, a pitch-derived needle coke SC incorporating oxygenated functional groups through oxidation by H_2_O_2_ (ONC-SC) was also reported to exhibit a high reversible capacity of 323 mAh g^−1^ at 50 mA g^−1^ compared with bare NC-SC (238 mAh g^−1^). At the optimal oxidation level, ONC-SC showed an expanded interlayer distance, abundant oxygenated functional groups, and defects on its surface, which serve as active sites for K^+^ storage and provide pathways for its migration [115].

*Hard carbon.* Inspired by early investigations in LIBs and NIBs, Ji’s group also pioneered the research on HC as an anode for KIBs. The potassiation/depotassiation profile of the HC microspheres they prepared consisted of two distinct regions: a high-voltage slope-shaped region and a low-voltage plateau-like region [116]. According to a later mechanism study, these regions are, respectively, associated with the absorption of K^+^ on the HC surface and the insertion/extraction process [117]. Compared with conventional graphite or SC, this mesoporous HC showed excellent cyclability, retaining approximately 83% of the capacity for 100 cycles. More importantly, the HC discharge plateau occurred at ~0.2 V (as in graphite), i.e., above K metal plating, so reducing the potential risk of dendrite formation. Favorably, the rate capability of HC can be greatly improved by adding conductive carbon to the electrode preparation, as Valma et al. pointed out [118].

One of the advantages of HC anode materials is that biomass could be used as a precursor, improving the reuse and recycling of this waste, which is usually burned in the open air, releasing volatile organic compounds and oxides into the atmosphere. Coconut shell-derived HC (CS-HC) has recently been reported to exhibit an excellent ICE of 87.32% and cycling performance, with a capacity retention of 92.8% after 100 cycles at 50 mA g^−1^, as well as good rate capability (~280 mAh g^−1^ at 300 mA g^−1^) due to its small specific surface area and expanded interlayer distance [119]. These results support using biowaste as an HC precursor to develop high-performance anode materials for KIBs.

**Figure 8:**
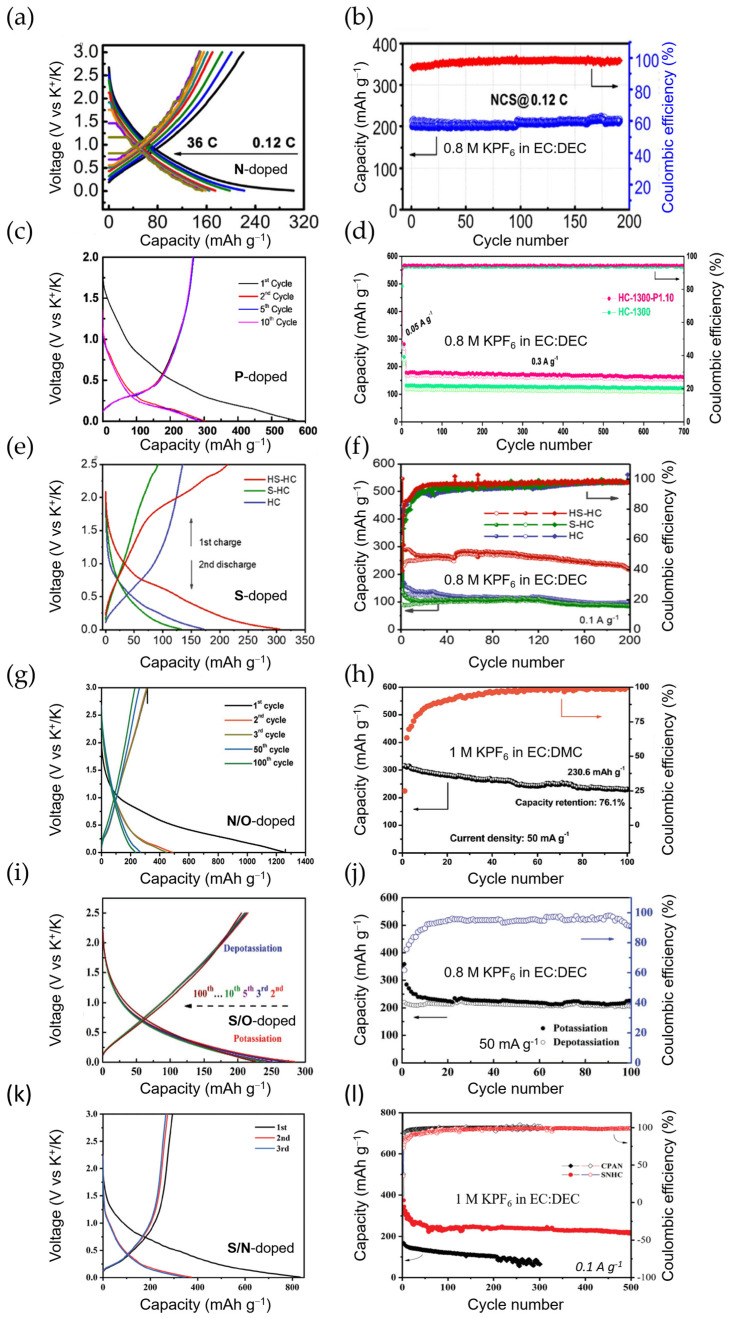
Voltage profiles and cycling performance of HC anode materials doped with (**a**,**b**) N (adapted from C. Chen [120]), (**c**,**d**) P (adapted from S. Alvin [122]), (**e**,**f**) S (adapted from Y. Zhang [123]), (**g**,**h**) N/O (adapted from J. Yang [124]), (**i**,**j**) S/O (adapted from M. Chen [125]), and (**k**,**l**) N/S (adapted from Y. Liu [126]), respectively. The electrolyte used and the current density applied are detailed in the cyclability graphs.

Similarly to other carbon-based materials, heteroatom doping has been adopted as a measure to upgrade the capacity, K^+^ diffusion rate, and ICE in HCs. So far, N-doping [120,121], P-doping [122], S-doping [123], and dual doping, such as N/O [124], S/O [125], and S/N [126], have been explored for HC anodes in KIBs. Their voltage profiles and cycling stabilities are shown in Figure 8. Lignite-derived HC, with a hierarchical porous carbon structure and doped with nitrogen, has recently shown good cyclic performance, delivering a reversible capacity of 314 mAh g^−1^ after 100 cycles at 0.1 A g^−1^ and 124.3 mAh g^−1^ after 1500 cycles at high current density (1.0 A g^−1^) [121]. However, probably the most impressive results have been reported for N-doped HC microspheres (N-doped CS, see Table 4 for further details) derived from chitin [120]. Through a sol-gel method and posterior carbonization, pyridinic N-rich (ca. 76%) CSs with hierarchical pores and an average diameter of 28 nm were synthesized. The N-doped CS exhibited an unprecedented high-rate capability of 154 mAh g^−1^ at 72C (~20.2 mA g^−1^) and an ultralong cycle stability (180 mAh g^−1^ at 1.8C) for 4000 cycles with no obvious capacity fading.

*Soft/hard carbon composites*. In 2023, an HC/SC hybrid carbon anode, in which HC domains (below 5 nm) were uniformly distributed in SC, was chemically synthesized by an esterification reaction. The synergistic effect of having both types of carbons enhanced the diffusion kinetics of K^+^ and the cycle life, exhibiting up to 121 mAh g^−1^ at the extremely high current density of 3.2 A g^−1^ and an average capacity decay of 0.078% per cycle at 1 A g^−1^ [127].

The electrochemical performance of most of the works addressed in this section dedicated to disordered carbons (SC and HC) are compiled in Table 4.

### 2.4. Other Carbonaceous Anode Materials

**Figure 9:**
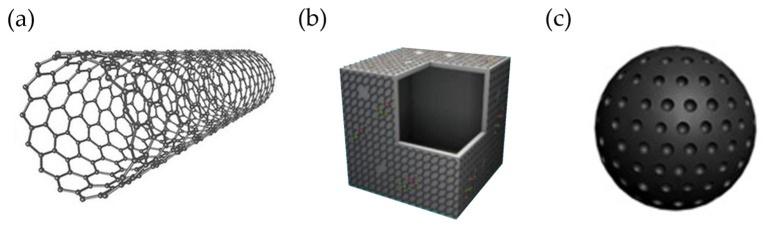
Typical structure of a (**a**) CNT (credit image: Mrs. Plugiano [128]), (**b**) CNC (adapted with permission from ref. [129]), and (**c**) CNS (reproduced with permission from ref. [130]).

Numerous nanostructured carbon materials, namely carbon nanofibers (CNFs), carbon nanotubes (CNTs), carbon nanocages (CNCs), and carbon nanospheres (CNSs), have been explored as anode materials for KIBs (see their structures in Figure 9). The main goal of their use is to lessen the volume expansion that a material undergoes with the insertion of K^+^ and thus prolong its cycle life.

One-dimensional (1D) materials, including nanofibers, nanotubes, and nanowires, are expected to show good rate performance in KIBs as a result of the shorter K ion diffusion distance as well as the interconnected conductive network.

*Carbon nanofibers.* The first insight into the electrochemical potassiation of individual hollow CNFs was conducted in 2014 by Wang et al., who observed a sloping profile. Despite the poor capacity retention attained, and we know now that this was due to the choice of electrolyte (1 M KPF_6_ in EC:DMC), the authors recognized CNFs as a viable material for KIBs [131]. Indeed, high capacity (272 mAh g^−1^ at 0.02 mA g^−1^), high-rate capability, and long cycle stability (0.01% capacity decay per cycle over 1200 cycles) were reported for flexible, free-standing CNF papers prepared by electrospinning, whose porous structure is believed to alleviate the volume expansion suffered during the insertion of K^+^ [132]. It is important to note, however, that a significant content of N and O heteroatoms, i.e., dual N/O doping, was found in these CNFs. Also benefiting from heteroatom doping, a superior rate capability and long cycle life have been displayed for K^+^ storage in CNFs (as shown in Table 5) [133,134,135]. Outstandingly, up to 146 mAh g^−1^ have been preserved after 4000 cycles at 2 A g^−1^, and around 100 mAh g^−1^ has been achieved at the high current density of 20 A g^−1^ [135].

*Carbon nanotubes.* SWCNTs (single-walled CNTs) and MWCNTs (multiwalled CNTs) were the first CNTs investigated as anode materials in KIBs back in 2017 [16]. However, although the electrochemical insertion of K^+^ was confirmed, releasing 196 mAh g^−1^ and 351 mAh g^−1^ at 5 mA g^−1^, respectively, both materials experienced a severe drop in capacity in the subsequent four cycles (prompted, from our point of view, by the electrolyte selection, which was 1 M KPF_6_ in EC:DMC). Shortly after, improved stability (91% capacity retention over 500 cycles) and high-rate performance were reported for free-standing hyperporous hierarchical MWCNTs. These hierarchical MWCNTs consisted of an inner CNT and an outer CNT, the former with densely stacked graphitic walls and the latter with more loosely stacked, disordered walls, which were interconnected to a hyperporous bulky sponge, conferring the material a unique structural stability [136]. Additionally, from hydrothermally synthesized carbon quantum dots (CQDs), porous carbon microtubes (PCMs) were obtained. With an interlayer spacing of 0.396 nm and doped with P and S elements, the PCMs maintained 260 out of ~350 mAh g^−1^ at 1 A g^−1^ after 100 cycles [137]. The K^+^ storage properties of N-doped CNTs [138], including self-standing N-doped CNTs [139], have also been evaluated and can be consulted in Table 5. Doping aside, a free-standing flexible CNTs/GCF anode with a 3D nanostructure, prepared by introducing CNTs into GCF (graphitic carbon foam), also led to excellent capacity retention and cycle lifespan. Of the 229 mAh g^−1^ delivered at 0.1 A g^−1^, 98% of the capacity (i.e., 226 mAh g^−1^) was preserved after 800 cycles, and 127 mAh g^−1^ (i.e., 96% capacity retention) over 2000 cycles at a higher current density of 0.5 A g^−1^ [140]. More information on CNTs reported in the last years as negative electrodes for KIBs can be found in the review [141].

*Carbon nanocages and carbon nanospheres.* In addition to CNFs and CNTs, the electrochemical performance of CNCs [142] and CNSs [143] in KIBs has been tackled (Table 5). Guo’s group fabricated graphitic CNCs by simple high-temperature treatment of Ketjen black at 2800 °C in an inert atmosphere. This material presented excellent cyclability, 195 mAh g^−1^ after 100 cycles (representing 92% of capacity retention) at 0.2C, and a superior rate capability of 175 mAh g^−1^ at 35C. Most likely, its hollow structure helps maintain structural integrity during cycling, and the hybrid K-storage mechanism, which combines faradaic (intercalation/deintercalation) and capacitive (surface adsorption/desorption) processes, would be responsible for the fast diffusion of K^+^; however, the ICE was only 40% [142]. On the other hand, S-grafted CNSs, containing O (3.4%) and being S-rich (37.6%), exhibited one of the highest reversible capacities (572 mAh g^−1^ at 25 mA g^−1^) reported in KIBs for a carbon-based material; and it had almost no capacity fade (ca. 150 mAh g^−1^) at 3 A g^−1^ for over 1000 cycles. Post-mortem Raman analysis revealed that S, either covalently bonded to carbon or nanoconfined within the carbon matrix, contributed to the capacity at these low voltages through a reversible secondary conversion reaction [143].

*Carbon nanosheets*. Recently, N-doped carbon nanosheets (N-doped CNsheets) have been obtained by freeze-drying a mixture of citric acid and urea in the presence of K_2_CO_3_ and by posterior annealing treatment at 750 °C. The resulting material exhibited an exceptional rate capability, delivering 468 mAh g^−1^ at 100 mA g^−1^, and outstanding lifespan of over 40,000 cycles at 2 A g^−1^ (see Table 5). Based on DFT calculations, the presence of suitable N dopants not only alleviates the volume change but also increases the electronic conductivity and enhances K^+^ adsorption [144].

*Less-rigid carbon materials.* In addition to nanostructured carbon anodes, many other, less rigid, carbonaceous materials have been developed for KIBs. Among them, some interesting examples include activated carbon (see Table 1) [78], mesoporous carbons [145], hollow carbons [146], and 2D and 3D carbons (see Table 5) [147,148].

For example, by combining short-range-ordered atoms of amorphous carbon, which have enlarged interlayer spacing that can better tolerate volume variations, and porous carbon, which increases the surface area, amorphous ordered mesoporous carbon (OMC) has been obtained. This amorphous OMC, whose interlayer spacing is estimated at 5.21 Å, has shown high endurance to volume variation (ca. 7%) during potassiation and depotassiation, resulting in a high capacity (up to 307 mAh g^−1^ at 0.05 A g^−1^), high-rate performance, as well as long cycle stability (147 mAh g^−1^ are still delivered over 1000 cycles at 1 A g^−1^) [145].

Structural engineering is another effective way to generate stable carbon anodes. Via simple pyrolysis of MF (melamine–formaldehyde) resin, neuron-like interconnected hollow carbon has been prepared, which can be used as a self-standing electrode. Its highly stable and flexible hollow structure, most likely induced by glass-blowing, ensures high electrochemical performance with superior cyclability, with no capacity fading observed over 150 cycles at 0.14 A g^−1^ [146]. On the other hand, 2D sheet-like carbon derived from a COF (covalent organic framework), with a carbon interlayer spacing of 0.4 nm, homogeneously co-doped with N and P, and rich in all types of pores (macro-, micro-, and mesopores), displayed a remarkably high capacity of 404 mAh g^−1^ at 100 mA g^−1^ and an excellent long-term cycle life (179 mAh g^−1^ at 1 A g^−1^ over 2000 cycles) [147]. Also, 3D amorphous carbon has been achieved after carbonization and activation in KOH of SAPs (superabsorbent polymers) from baby diapers. With a porous nanostructure and short-ranged graphitic domains with an increased interlayer spacing (4.09 Å), this material exhibited a high capacity (430 mAh g^−1^ at 0.05 A g^−1^) and was capable of retaining 162 mAh g^−1^ after 1000 cycles at 1 A g^−1^ [148].

Table 5 shows the electrochemical properties extracted from some works tackled in this section dedicated to other carbonaceous materials (specifically, CNFs, CNTs, CNCs, CNSs, CNsheets, and less-rigid carbon materials).

If we notice, heteroatom doping is a common strategy used for carbon materials intended for KIBs. Their ameliorated performance by heteroatom doping can be summarized as follows. Indistinctly, N- or S-doping increases the conductivity and offers additional capacitive capacity. Doping with B- or N-, however, enhances the K^+^ absorption energy of carbon. The introduction of N-, S-, or P-heteroatoms enlarges the interlayer distance. On the other hand, P- or S-doping is conducive to (conversion) reactions with K^+^, which contributes to boosting capacity. Dual- or triple-doping brings together the respective advantages of the involved heteroatoms to further enhance the K^+^ storage properties. Nonetheless, the impact of doping positions (either between layers or replacing some atoms in the carbon network) and the cooperative effects of multiple heteroatoms doping on the K-ion storage performance should be inspected in detail.

**Table 5:** Electrochemical performance of other carbonaceous anode materials in KIBs.

					Cyclability		Rate Capability	
Anode Material	Electrolyte	Binder	ICE	Initial Capacity (mAh g^−1^)@ Current Density (A g^−1^)	Capacity (mAh g^−1^)	Cycle Number@ Current Density (A g^−1^)	Capacity (mAh g^−1^)@ Current Density (A g^−1^)	Ref.
S-s. CNF paper ^1^ (N/O-doped)	0.8 M KPF_6_/EC:DEC	Free	24.1%	272@0.2 223@0.2	270 211	80 cycles@0.02 1200 cycles@0.2	100@7.7	[132]
S-s. N/O-doped CNF ^1^	0.8 M KPF_6_/EC:DEC	Free	35%	~280@0.028 ~190@0.28	170	1900 cycles@0.28	~120@1.4	[133]
N-doped (chitin-d) ^2^ CNF	0.8 M KPF_6_/EC:DEC	Na-alginate	37.8%	215@0.056	~200 103	100 cycles@0.056 500 cycles@0.56	~85 @1.4	[134]
N-doped CNF	0.8 M KPF_6_/EC:PC	CMC	49%	368@0.025	248 146	100 cycles@0.25 4000 cycles@2	101@20	[135]
S-s. Hierarchical MWCNT ^1^	0.8 M KPF_6_/EC:DEC	Free	15%	232@0.1	210	500 cycles@0.1	162@1.6	[136]
Carbon MICROtube (P/S-doped)	0.8 M KPF_6_/EC:DEC + 3 wt.% FEC	PVDF	42.5%	450@0.5 N/A ^3^	395 176	100 cycles@0.5 2000 cycles@2	177@1	[137]
N-doped CNTs	0.8 M KPF_6_/EC:DEC	PDVD	23.3%	380@0.5	204	1000 cycles@0.5	N/A	[138]
S-s. N-doped CNTs ^1^	N/A	Free	14%	324@0.02	236	100 cycles@0.02	75@1	[139]
CNTs/GCF	0.7 M KPF_6_/EC:DEC	Free	24%	229@0.1 132@0.5	226 127	800 cycles@0.1 2000 cycles@0.5	~75@1	[140]
Graphitic CNCs	1 M KFSI/EC:PC	CMC+PAA	40%	212@0.056	195	100 cycles@0.056	175@9.8	[142]
S-grafted CNSs	0.8 M KPF_6_/EC:DEC	PVDF	51.4%	572@0.25 160@3	~290 ~150	250 cycles@0.2 1000 cycles@3	110@5	[143]
N-doped CNsheets	3 M KFSI/DME	CMC	~34%	420@0.1	~370 ~140	100 cycles@0.1 40,000 cycles@2	~185@2	[144]
Amorphous OMC	0.8 M KPF_6_/EC:DEC	PVDF	63.6%	307.4@0.05 ~175@1	257 147	100 cycles@0.05 1000 cycles@1	114@0.4	[145]
S-s. Hollow carbon ^1^ (neuron-like)	0.8 M KPF_6_/EC:DEC	Free	72.1%	340@0.028	250 ~115	150 cycles@0.14 ~175 cycles@0.28	~115@0.56	[146]
2D Sheet-like carbon (N, P-doped)	0.8 M KPF_6_/EC:DEC	PVDF	49%	404@0.1 250@1	350 179	300 cycles@0.1 2000 cycles@1	90@5	[147]
3D carbon	0.8 M KPF_6_/EC:DEC	CMC	23.6%	430@0.05 ~185@1	270 162	100 cycles@0.05 2000 cycles@1	78@5	[148]

^1^ S-s. = self-standing. ^2^ chitin-d = chitin-derived. ^3^ N/A = not available.

In brief, great effort has been devoted to improving graphite’s limitations (structural integrity during charge/discharge cycles, K^+^ conductivity, etc.), and many carbon-based materials are being considered as alternatives to graphite. Graphene and disordered materials, such as hard and soft carbons, present a slope-shaped voltage profile and a higher average working potential than graphite, thus leading to a lower risk of dendrite formation but also to a lower full-cell energy density. In general, hard carbons achieve superior cyclability (up to 4000 cycles have been reported) since their structures can tolerate the volume variations induced by K^+^ insertion much better, and soft carbons possess excellent rate capabilities. Alternatively, carbonaceous materials, whether nanostructured or not, typically exhibit both long-term stability and high-rate performance. Nevertheless, although similar or even higher specific capacities (above 450 mA h g^−1^ in some graphene-derived and non-nanostructured carbons) have been reached with these materials, their volumetric energies (as a result of the lower bulk densities) and ICE are inferior, and their production costs increase compared with graphite. Taking all this into account, some of the future directions to pursue could be to develop simple and inexpensive synthetic methodologies and improve the ICE. To overcome this last challenge, as we have seen for graphite, the optimization of the electrolyte and binder, SEI preformation (artificial SEI), as well as pre-potassiation are effective strategies that should be adopted. Further morphological modification, compositional tuning, and surface engineering may be explored. Meanwhile, a deeper understanding of the influence that the carbon structure has on the K^+^ storage mechanism is urgently needed.

## 3. Other Intercalation Anode Materials

### 3.1. Titanium Oxides

Titanium-based oxide anodes have numerous merits, such as fabulous chemical and thermal stability, non-toxicity, and abundance. They also present a higher average working voltage than graphite (typically between 0.6 and 1 V vs. K^+^/K), making them safer anodes. On the other hand, they exhibit poor electrical conductivity, which theoretically limits their applications in KIBs [149]. In fact, compared with their LIB and SIB counterparts, relatively little research has been conducted on titanium-based anodes in KIBs.

**Figure 10:**
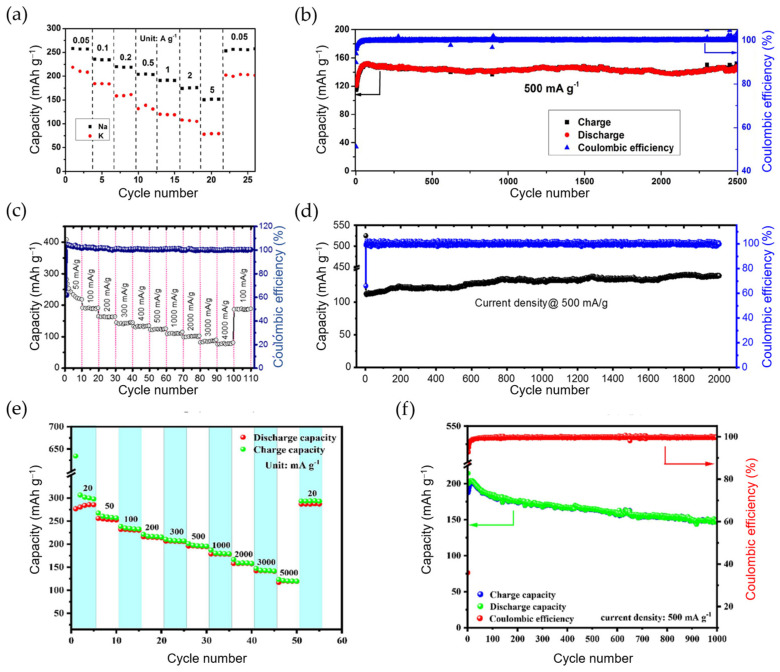
C-rate capabilities and long-term cyclabilities of (**a**,**b**) TiO_2_-coated layered titanate with polyaniline intercalated, HTO-PANI-600 (reproduced with permission from J. Liao [151]), (**c**,**d**) TiO_2_@NGC (reproduced with permission from J. P. Dubal [153]), and (**e**,**f**) Mn_0.5_Ti_2_(PO_4_)_3_@C (reproduced with permission from S. Liu [164]).

The most representative titanium oxide material is TiO_2_. However, its low ion/electron conductivity, along with the slower ionic diffusion and larger ionic radius of K^+^, drastically limits its K storage. Although simple nanostructuring might be a possible solution to overcome this issue, it remains a challenge. With the aim of improving the electrical conductivity, structural modifications and/or carbon incorporation have been implemented to obtain hierarchical tubular TiO_2_-carbon heterostructure (HeTiO_2_eC) microtubes [150], TiO_2_-coated layered titanate with polyaniline intercalated (see Figure 10a,b) [151], G-TiO_2_ (graphene coatings on the surface of TiO_2_ nanotubes) [152], TiO_2_@NGC (TiO_2_ nanoparticles (NPs) encapsulated in N-rich graphitic porous carbon) (see Figure 10c,d) [153], carbon-coated flower-like TiO_2_ nanospheres [154], and sandwich-like structured TiO_2_/graphene composites [155], which showed superior K storage performances (see Table 6). Interestingly, the incorporation of MWCNTs on anatase TiO_2_ to form a TiO_2_/CNT composite results in the irreversible transformation of TiO_2_ into the Magnéli phase, Ti_6_O_11_, after the first insertion/deinsertion of K. In the following cycles, via conversion reaction, this Magnéli Ti_6_O_11_ phase reversibly evolves toward the discharge products Ti_4.5_O_5_ (reduced phase) and K_6_Ti_2_O_7_ (K ion-rich phase), delivering a specific capacity of ~150 mAh g^−1^ at 0.05 A g^−1^ [156]. Recently, He’s group reported using Ti_3_C_2_/TiO_2_/rGO nanosheets as high-stable anodes for KIBs, where TiO_2_ NPs were formed in situ after calcination of Mxene Ti_3_C_2_. The synergistic effect of Ti_3_C_2_ (a strong electrical conductor with a high specific surface and chemically active area), rGO (a substrate with high surface area as well), and TiO_2_ (which upon entering within the Ti_3_C_2_ increases its interlayer spacing and prevents its structural collapse), efficiently buffers volume variations and provides a rapid transport pathway for electrons and ions. Consequently, Ti_3_C_2_/TiO_2_/rGO exhibits a good cycle stability and electrochemical performance at relatively high current densities (see Table 6) [157].

Heteroatom doping has also been deployed to prepare Ta-doped TiO_2_/CNFs [158] and 3D F-doped TiO_2_ nanorods [159], the latter of which has allowed the development of dendrite-free metal anodes, including K.

K_2_Ti_4_O_9_ has a layered structure that can accommodate up to 2 K^+^ per formula unit, according to the following reversible reaction: K_2_Ti_4_O_9_ + 2 K^+^ + 2 e^−^ ⇔ K_4_Ti_4_O_9_. Kishore et al. synthesized this phase using a solid-state method and reported a specific capacity of 80 mAh g^−1^ at 0.1 A g^−1^ for K^+^ intercalation [160]. Better results were attained with ultrathin K_2_Ti_4_O_9_ nanoribbons (M-KTO) prepared by concomitant oxidation and alkalization of Ti_3_C_2_ MXene nanosheets. Benefiting from the 0.93 nm interlayer and its microporous structure, M-KTO showed more than double the capacity reported by Kishore and an extended cycle life (88 mAh g^−1^ at 0.3 A g^−1^ over 900 cycles) [161].

K_2_Ti_8_O_17_ is another attractive Ti-based layered-structure oxide, with open channels suitable for K^+^ transport and storage and a theoretical capacity (as long as all the Ti^4+^ is reduced to Ti^3+^) of 308 mAh g^−1^. Xu’s group first reported its utilization as an anode for KIBs. Through a hydrothermal synthesis followed by an annealing process, they obtained acanthosphere-like K_2_Ti_8_O_17_ nanorods with an interlayer distance of 0.367 nm capable of delivering 182 mAh g^−1^ at 20 mA g^−1^ [162].

Xu’s group also investigated the K^+^ storage performance of KTi_2_(PO_4_)_3_. Using the same methodology as for K_2_Ti_8_O_17_, KTi_2_(PO_4_)_3_ nanocubes with an interlayer spacing of 0.367 nm and a 3D framework were prepared [163]. Inferior capacities but better ICE than K_2_Ti_8_O_17_ were found for KTi_2_(PO_4_)_3_. Also, the beneficial effect of carbon-coating KTi_2_(PO_4_)_3_ (referred to as KTi_2_(PO_4_)_3_ nanocubes@C) on its cycle stability was evidenced. Meanwhile, Liu and coworkers proposed a Mn-doped Ti-based NASICON structure Mn_0.5_Ti_2_(PO_4_)_3_ polyanion compound embedded in 3D carbon microspheres to develop an anode material with a stable structural framework, large ion channels, fast ion mobility, and enhanced electronic conductivity. The Mn_0.5_Ti_2_(PO_4_)_3_@C exhibits high rate capability, delivering 306 and 123 mAh g^−1^ at 20 and 5000 mA g^−1^, respectively, with excellent cycling stability (148 mAh g^−1^ at 500 mA g^−1^ after 1000 cycles), as shown in Figure 10e,f) [164].

Other phosphate derivatives have also been reported, including KTiOPO_4_ [165] and KTiPO_4_F [166]. KTiOPO_4_ possesses an average plateau of ca. 0.8 V vs. K^+^/K, which is ca. 0.33 V below that of KTi_2_(PO_4_)_3_, so elevating the full-cell voltage but still preventing K-dendrite formation. It can achieve 102 mAh g^−1^ at 5 mA g^−1^ by reversible (de)intercalation of ~0.75 K^+^ and retain 80% of the capacity after 50 cycles. Furthermore, it benefits from a quasi-3D expansion, i.e., an expansion in the three directions of the space, with a cell volume expansion of 9.5% comparable to that observed for graphite in LIBs, which ensures structural stability [165]. Simply reducing the size to the nanoparticle range, which shortens the K^+^ diffusion pathway and stimulates its de(intercalation), high capacity (192 mAh g^−1^ at 30 mA g^−1^), long life (78% capacity retention after 10,000 cycles at 3 A g^−1^), high rate (100 and 84 mAh g^−1^, respectively, at 1.5 and 3 A g^−1^), as well as a reasonable capacity at low-temperature operation (62 mAh g^−1^ at −5 °C) have been reported for KTiOPO_4_ obtained by one-step low-temperature hydrothermal synthesis in H_2_O: EtOH 1: 7 *v/v* [167]. Also through facile hydrothermal synthesis, KTiPO_4_F has been prepared, although additional coating with carbon and mixing with graphene platelets was implemented prior to evaluating it as an anode for KIBs [166]. With a similar voltage plateau as KTiOPO_4_ (0.8 V vs. K^+^/K), KTiPO_4_F is capable of delivering 205 mAh g^−1^ at C/5 (26 mA g^−1^), while only 130 mAh g^−1^ are maintained after 100 cycles. Positively, the cell volume variation has been calculated to be only 8.5%, positioning it as the lowest among its anode competitors, according to the authors of the investigation.

For their part, oriented K_2_Ti_6_O_13_ nanorods coated with a 4–11 nm carbon layer have shown around 150 mAh g^−1^ at 25 mA g^−1^, with good capacity retention (approx. 80% after 200 cycles) and improved K^+^ diffusion and electronic conductivity [168].

Table 6 gathers the K storage activity of most of the studies mentioned in Section 3.1, dedicated to titanium oxides.

**Table 6:** Electrochemical performance of titanium oxides as anode materials in KIBs. N/A stands for not available.

					Cyclability		Rate Capability	
Anode Material	Electrolyte	Binder	ICE	Initial Capacity (mAh g^−1^)@ Current Density (A g^−1^)	Capacity (mAh g^−1^)	Cycle Number@ Current Density (A g^−1^)	Capacity (mAh g^−1^)@ Current Density (A g^−1^)	Ref.
HeTiO_2_eC microtubes	0.8 M KPF_6_/EC:DEC	CMC	49.1%	241@0.1 163@0.5	197 133	200 cycles@0.1 1200 cycles@0.5	97@2	[150]
TiO_2_-coated polyaniline	3 M KFSI/DME	CMC	56.6%	219@0.05 110@0.5	198 150	400 cycles@0.05 2500 cycles@0.5	80@5	[151]
G-TiO_2_	0.8 M KPF_6_/EC:DMC	Na-alginate	39%	320@0.05 129@5	222 96	400 cycles@0.1 3000 cycles@5	129@5	[152]
TiO_2_@NGC	1 M KPF_6_/EC:DMC	PVDF	44%	228@0.05 ~120@0.5	~270 185	100 cycles@0.05 2000 cycles@0.5	114@1	[153]
C-coated flower-like TiO_2_	3 M KFSI/DME	Na-alginate	32%	172@0.036 ~100@0.36	137 ~100	100 cycles@0.036 2500 cycles@0.36	93@0.7	[154]
TiO_2_/graphene composite	0.8 M KPF_6_/EC:DMC	PVDF	42%	337@0.1	245	100 cycles@0.1	174@0.6	[155]
Ti_3_C_2_/TiO_2_/rGO	0.8 M KPF_6_/EC:DEC	PVDF	20.3%	487@0.1 294@0.5	349 229	200 cycles@0.1 500 cycles@0.5	222@1	[157]
Ta-doped TiO_2_/CNFs	1 M KFSI/EC:DMC	PVDF	37.9%	255@0.05 122@2	N/A 149	N/A 800 cycles@2	103@5	[158]
K_2_Ti_4_O_9_	1 M KPF_6_/EC:PC	PVDF	~20%	~80@0.1	~40	30 cycles@0.1	50@1	[160]
M-KTO (KTO = K_2_Ti_4_O_9_)	1 M KPF_6_/DEGDME	PVDF	25.9%	151@0.05 120@0.2	~92 50	100 cycles@0.05 900 cycles@0.2	81@0.3	[161]
K_2_Ti_8_O_17_ nanorods	0.8 M KPF_6_/EC:DMC	PVDF	~66%	182@0.02	102	50 cycles@0.02	44@0.5	[162]
KTi_2_(PO_4)3_ nanocubes	0.8 M KPF_6_/EC:DEC	PVDF	~92%	~68@0.064	~30	100 cycles@0.064	N/A	[163]
KTi_2_(PO_4)3_ nanocubes@C	0.8 M KPF_6_/EC:DEC	PVDF	~78%	~60@0.064	~80	100 cycles@0.064	N/A	[163]
Mn_0.5_Ti_2_(PO_4_)_3_@C	1 M KFSI/EC:DEC	PVDF	43.6%	276@0.02 186@0.5	236 148	200 cycles@0.1 1000 cycles@0.5	~125@5	[164]
KTiOPO_4_	5 M KFSI/DEGME	CMC	66%	102@0.005	~82	50 cycles@0.005	N/A	[165]
KTiOPO_4_ NPs	0.8 M KPF_6_/EC:DEC	CMC	N/A	161@0.15 84@3	139 66	100 cycles@0.15 10,000 cycles@3	84@3	[167]
KTiPO_4_F@C + G nanoplates	1 M KPF_6_/EC:PC	PVDF	~60%	~205@0.026 N/A@0.13	133 130	100 cycles@0.026 1000 cycles@0.13	50@1	[166]
K_2_Ti_6_O_13_@C	1 M KFSI/EC:DEC	PVDF	~25%	151@0.025	119	200 cycles@0.025	65@0.5	[168]

### 3.2. Vanadium Oxides

Analogously to titanium-based oxides, vanadium-based oxides present a layered structure with extra space suitable for accommodating K^+^. Additionally, the multivalency of vanadium, whose oxidation states range from V^2+^ to V^5+^, offers the possibility of further expanding the theoretical capacity, making V-based compounds attractive as potential negative materials for KIBs [169].

**Figure 11:**
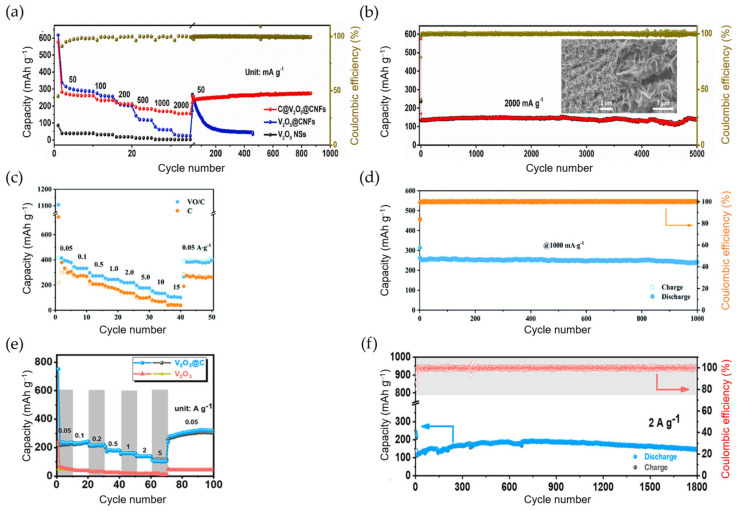
C-rate capabilities and long-term cyclabilities of (**a**,**b**) C@V_2_O_5_@CNFs (reproduced with permission from X. Xiang [171]), (**c**,**d**) VO@C (reproduced with permission from J. Lu [172]), and (**e**,**f**) V_2_O_3_@C (reproduced with permission from J. Hu [178]).

The first vanadate evaluated in KIBs was the flower-like-shaped K_0.23_V_2_O_5_. Luo’s group obtained it via the hydrothermal method followed by calcination. The material showed a high initial specific capacity, 404 mAh g^−1^ at 20 mA g^−1^, but maintained only 121.6 mAh g^−1^ (approx. 30% capacity retention) after 150 cycles [170]. Improved results have been attained with free-standing carbon-coated V_2_O_5_ nanosheets arranged on CNFs (C@V_2_O_5_@CNFs), recently reported by Zhou et al. as a superior vanadium-based anode for KIBs (Figure 11a,b). CNFs help prevent V_2_O_5_ self-stacking and the carbon coating stabilizes the structure and enhances the electrical conductivity. Therefore, C@V_2_O_5_@CNFs can maintain a high capacity (139 mAh g^−1^) after 5000 cycles at 2 A g^−1^ [171].

On the other hand, a robust spring-like lamellar nanostructure embedding VO NPs into amorphous carbon (VO@C) has been designed to accommodate the volume change during K storage. Based on DFT calculations, this structure increases the electronic states at the Fermi level, leading to an improved ionic conductivity and to a reduction in the K^+^ diffusion barrier, thus contributing to achieving a large capacity and superior rate performance. In fact, VO@C exhibits a superb stability (241 mAh g^−1^ at 1 A g^−1^ over 1000 cycles) and rate performance (104 mAh g^−1^ at 15 A g^−1^), as shown in Figure 11c,d [172].

Layered K_2_V_3_O_8_, synthesized by a facile hydrothermal method, has also been tested as a novel anode material for K intercalation [173]. Although K_2_V_3_O_8_ exhibited relatively high initial capacity (282 mAh g^−1^ and 270 mAh g^−1^ at 50 and 100 mA g^−1^, respectively), severe capacity degradation was observed over the cycles, with only ca. 31.1% capacity retention after 180 cycles at 0.1 A g^−1^. However, since this capacity decay was attributed to side reactions between the electrolyte and K metal, better results could be attained with another electrolyte and in a full-cell configuration.

Among vanadium oxides, VO_2_ has a bilayer structure with large interlayer spacing, which facilitates the reversible insertion/extraction of K^+^ and endures the resulting volume expansion. Nonetheless, slow K^+^ diffusion kinetics and poor electronic conductivity are its main limitations. To overcome these drawbacks, Zhang’s group engineered VO_2_ nanorods with an amorphous surface (SA-VO_2_) through a facile hydrothermal reaction and subsequent chemical reduction. The intimate interaction between the crystalline VO_2_ core and its oxygen-defective amorphous shell creates intimate heterointerfaces that can accelerate the interfacial charge transfer and additional active sites for K^+^ storage. Therefore, large capacity (290 mAh g^−1^ at 50 mA g^−1^), good rate capability (179 and 141 mAh g^−1^, respectively, at 1 and 2 A g^−1^) and impressive cycle stability (86% capacity retention after 500 cycles at 0.5 A g^−1^) were attained with this material [174].

Motivated by this interfacial engineering, Gao’s group recently designed a VO_2_-V_2_O_5_ composite embedded in a 3D N-doped carbon matrix (denoted as VO_2_-V_2_O_5_/NC). Again, as a result of the favorable interfacial effect between the ultrasmall size of the VO_2_-V_2_O_5_ heterostructures, but also due to the highly conductive 3D carbon network, a distinctive K-ion storage is achieved, exhibiting high capacity, significant long-term stability (501 and 256 mAh g^−1^ are maintained after 120 and 1600 cycles at 0.1 and 1 A g^−1^, respectively), and high-rate capability (108 mAh g^−1^ at 10 mA g^−1^) [175].

Another promising anode material for KIBs, due to its 3D open-tunnel structure, is V_2_O_3_. However, it faces similar challenges to its counterpart VO_2_, i.e., low electron/ion conductivity and, as a result of the substantial volume variation experienced during the K^+^ intercalation/deintercalation process, a fast capacity decay. To address these disadvantages, Jin et al. fabricated flexible self-standing V_2_O_3_@PNCNF electrodes by embedding V_2_O_3_ NPs into N-doped 1D porous CNFs via electrospinning and posterior annealing. Evaluation of V_2_O_3_@PNCNFs as anodes for K^+^ intercalation resulted in a reversible capacity of 240 mAh g^−1^ at 50 mA g^−1^, excellent capacity retention of ca. 96% after 500 elapsed cycles, and fast charge/discharge capability, still delivering 134 mAh g^−1^ at 1 A g^−1^ [176]. Enhanced capacity was achieved with carbon-coated V_2_O_3_ hollow spheres (HS-V_2_O_3_@C) synthesized by a solvothermal reaction. The homogeneous carbon coating improves the electronic conductivity, and the hollow structure buffers the volume variations upon cycling, resulting in a superior performance. Consequently, it can retain 330 mA h g^−1^ at 100 mA g^−1^ after 500 cycles and 79 mA h g^−1^ at a high current density of 5000 mA g^−1^ [177]. Furthermore, to overcome the limitations of V_2_O_3_, Hu et al. encapsulated V_2_O_3_ NPs in amorphous carbon nanosheets (V_2_O_3_@C) [178]. On the one hand, the nanometric size of the V_2_O_3_ shortens the ion/electron diffusion paths and ameliorates the electrochemical reactivity. On the other hand, carbon nanosheets increment the tolerance to volume change and the contact area between the electrolyte and the active materials. Furthermore, the structure is strengthened, and charge transfer is accelerated across the composite interface, since C-O-V bonds are formed. Consequently, V_2_O_3_@C displayed high C-rate performance, exhibiting 117 mAh g^−1^ at 5 A g^−1^ and long cyclability, retaining ca. 150 mAh g^−1^ over 1800 cycles at 2 A g^−1^, as Figure 11e,f illustrate.

More details related to the K-intercalation properties of most of the studies on vanadium-based oxides mentioned in this section are summarized in Table 7.

From the above, it can be concluded that despite the strengths of titanium- and vanadium-based materials (i.e., safety and structural stability), their relatively low reversible capacity and poor electrical conductivity have hampered their development in KIBs. In the case of titanium-based anode materials, although the hydrothermal route (combined or not with sintering) is adequate for their synthesis, other novel, simple, and low-cost methods should be explored to better control the morphology, particle size, and porosity, thus promoting ion/electron diffusion and extending their cycle life. In the case of vanadium-based compounds, although the initial capacities delivered are acceptable, they suffer a drastic capacity decay. The best results, in terms of stability, were obtained with V_2_O_5_, VO, or V_2_O_3_, but the C-coating/N-doping is crucial. In general, strategies such as carbon coating, doping, size reduction, or the formation of carbon-based composites have proven to help enhance K-storage performance, thereby overcoming the low electrical conductivity of titanium- and vanadium-derived anode materials. As for boosting their relatively low reversible capacity, high-capacity active materials can be incorporated, thus forming composites.

## 4. Conclusions and Perspectives

This review emphasizes the potential of KIBs for several applications, such as EVs and large-scale stationary applications. The abundance and global distribution of potassium, as well as the lack of critical raw materials in their electrode components, unlike LIBs, make KIBs as sustainable and low-cost as NIBs. Advantageously, KIBs are more attractive for high-power applications than NIBs due to the faster diffusion of K^+^ into liquid electrolytes. In addition, if the obstacle of K^+^ diffusion in solids is overcome and an optimal electrode composition and electrode–electrolyte configuration designed, high energy density and long-term KIBs could be achieved that will be competitive with LIBs due to the proximity in their standard reduction potentials. Nevertheless, KIBs are still in their infancy, and more investigations need to be conducted to understand how their electrochemical performances could be improved to make their commercialization a reality.

The main challenges are related to the electrochemical properties of the anode materials and their reactivity and compatibility with a liquid electrolyte. Although great achievements have been made in the development of anode materials for KIBs, some of their issues (e.g., improving the ICE, specific capacity, long-term stability, etc.) still need to be overcome before moving from the lab scale to the prototype or industrial level. Particularly, this work has comprehensively reviewed recently reported intercalation anode materials for KIBs, such as carbon-based species and titanium- and vanadium-based oxides, with the aim of opening new avenues of research and developing competitive anode materials and, in turn, competitive KIBs.

Carbon-based materials are the best candidates as anode electrodes for KIBs—as are graphite and hard carbon, respectively, for LIBs and NIBs—mainly due to their low potassiation voltage, easy synthetic route, and flexibility to tune physicochemical and electrochemical properties during their fabrication. Among them, graphite seems to stand as the potential winning horse, but this remains to be proven. Therefore, graphite, soft-carbon and hard-carbon materials are primarily the only viable options for KIBs. However, they still present a great variety of challenges and issues and are far from perfect anode materials.

Regarding synthesis, the manufacture of carbon materials, such as graphite and soft carbon, requires high temperatures (≥2500 °C) and low heating rates (0.1–5 K min^−1^), significantly increasing energy consumption and, consequently, the production cost [19,108,179]. Hard carbon could represent a better choice since it can be obtained at lower temperatures (<1200 °C). However, its synthetic yield is usually less than 30%, and although the use of biowaste as a precursor could reduce the market price, its chemical properties have a direct influence on the final electrochemical properties of hard carbon [180]. Therefore, new and cost-effective manufacturing methods must be designed to make carbon-based anode materials good candidates in terms of production cost.

As just mentioned, another important aspect is that the final properties of soft- and hard-carbon anode materials depend on a wide variety of parameters, such as the source and properties of the precursor, the pre- and post-treatment steps, the annealing conditions (temperature, time, heating rate, inert atmosphere and its flow), etc. In other words, there is no standard synthesis protocol for soft- and hard-carbon anode materials.

In addition, the K^+^ storage mechanism remains controversial. In fact, several mechanisms have been proposed for graphite and hard-carbon anodes in the last years, making it difficult to understand which properties are crucial for enhancing K^+^ storage, although again, these are related to the fact that each investigation uses carbon anodes with intrinsically different properties. Hence, it is not possible to extrapolate the results obtained with one type of carbon to other carbon-based materials, making it difficult to standardize synthetic production protocols.

Moreover, techniques for characterizing carbon microstructures, pore properties, and K-storage mechanisms are still limited, further complicating the standardization of carbon manufacturing. Therefore, not only should material-level studies be performed to better understand the process, but additional techniques or new measurement protocols should be developed to understand and characterize the carbon-based material well.

Challenges related to the electrochemical properties of graphite and soft- and hard-carbon anode materials, in general, are their low ICE, limited specific capacity, poor rate capability and cycling stability, and large volume expansion upon (de)potassiation. Although in recent years multiple strategies have been reported with excellent results, they are still insufficient to achieve performances similar to the graphite or hard carbon in LIBs and NIBs, respectively. The main approaches are related to electrode and electrolyte engineering, such as heteroatom doping, control of particle size and shape, surface modifications, and adjustment of the chemistry and/or formulation of the electrode and/or electrolytes.

For example, heteroatom doping is commonly used to enhance the K^+^ storage capacity by enlarging the interlayer distance of graphite or by creating absorption sites on the surface of the carbon anode and thus enhancing its electronic conductivity. Nonetheless, as mentioned in the review, the impact of the doping positions and cooperative effects of multi-heteroatom doping on K ion storage performance need to be examined in detail. Indeed, the addition of heteroatom(s) usually induces a large specific surface area on the carbon anode, significantly increasing the side reactions between the active material and the electrolyte, and thus, drastically reducing its reversibility (and affecting its ICE) [180]. Therefore, in the heteroatom doping strategy, an equilibrated balance between doping and the specific surface area formed is critical.

Another possible strategy to boost the specific capacity, as well as rate performance and cycle stability, is the use of 1D (CNTs, CNCs, CNFs, etc.) and 2D (graphene) carbon-based materials. For instance, carbon nanonets have been successfully used to enhance the electrochemical properties of MoS_2_. By embedding MoS_2_/C into a 3D network derived from biomass, a MoS_2_/C@CNs composite is obtained, which retains 350 mAh g^−1^ at 200 mA g^−1^ after 400 cycles, and 90% of its initial capacity after 1000 cycles at 2 A g^−1^ [181]. However, these 1D and 2D materials are not an alternative for real applications due to their very low bulk density (even lower than that of hard carbon), which leads to poor volumetric energy densities. In our opinion, these 1D and 2D carbon-based materials would be best used in electrode composites, which could be beneficial, for instance, to increase the electronic conductivity or behave as a matrix to limit and retain the volume expansion upon cycling.

The benefits of using 1D and 2D carbon-based materials for composites with titanium- and vanadium-based oxides have been reported as well. Titanium-based compounds (vanadium-based materials are less attractive in terms of sustainability and toxicity) are often coated and/or composed with 1D/2D carbons to enhance their poor electronic conductivity. For example, the KTiNbO_5_/rGO nanocomposite delivered a reversible charge capacity of 128 mAh g^−1^ at 20 mA g^−1^, while in pure KTiNbO_5_, the charge capacity dropped to 66.7 mAh g^−1^ at the same current [182]. Nevertheless, although their applications in KIBs are currently limited due to their low conductivity and restricted K^+^ diffusion, these non-carbonaceous intercalation anode materials should be seriously considered in the near future, as they show excellent chemical and thermal stability, non-toxicity, and abundance, and they exhibit higher average operating voltages than carbon-based anodes, making them safer anodes.

On the other hand, although alloy- and conversion-type materials (not addressed in this review) could be considered second-generation anode electrodes due to their higher specific theoretical capacities, the reality is that they exhibit significant capacity loss upon cycling caused by (i) a large volume expansion, resulting in aggregation and pulverization of the active material and (ii) formation of an unstable SEI, newly exposing its surface to additional decomposition reactions (during the charge/discharge processes). Considering the larger ionic radius of the K ion, these types of anode materials are not an option (at least at this stage) for KIBs. Nonetheless, like in LIBs, their composites with graphite (the latter in a dominant proportion) could be an attractive solution.

Moving back to the most promising anode materials, graphite and soft and hard carbon, their ICE, reversible capacity, and cycle stability can be controlled by electrode and electrolyte engineering.

On the one hand, the electrode composition and surface engineering are crucial because they could block the degradation of the electrode surface resulting from contact with the electrolyte. The binder, which is usually considered an inert component of electrodes, contributes to the SEI formation and, in turn, to the first reversibility (i.e., ICE). It is not yet clear, but aqueous binders, such as CMC, could participate in the formation of SEI due to its decomposition upon reduction, protecting the surface of the carbon-based electrode from the first cycle, in addition to being beneficial in terms of sustainability in the electrode production. Another approach to improving the ICE and, consequently, its reversible capacity and long-term stability, could be the creation of some protective surface coating and/or resorting to an artificial SEI that could prevent the electrode degradation and tune the reaction of decomposition of the electrolyte with the electrode and, in turn, SEI formation.

On the other hand, the selection of electrolyte is another critical parameter, as already demonstrated for LIBs and NIBs. In the case of LIBs, the addition of the EC co-solvent, which prevents graphite exfoliation during cycling and helps form a stable SEI, was crucial for their commercialization in 1991 [183]. In NIBs, although mainly carbonate-based electrolytes are used, non-carbonate-based electrolytes have been reported to be better candidates because they do not form carbonate species in the SEI, which are highly soluble in the electrolyte, providing greater stability upon cycling [184,185]. In the case of KIBs, the presence of carbonate-based species, either as solvents or additives such as FEC, lead to not so favorable (or even detrimental) electrochemical performance. Additionally, the standard KPF_6_ salt is not the best choice since, as already mentioned in this review, it does not form enough inorganic species upon reduction to stabilize the K-based SEI. Ether-based and KFSI salt-based electrolytes are one possible solution. However, they are known to exhibit poor oxidation stability, also causing corrosion of the Al current collector at high potential; being, in general, incompatible with high-voltage cathodes; and hampering the development of high-energy-density KIBs. Although highly concentrated KFSI-based electrolytes ameliorate the corrosion stability of aluminum, this comes at a price (literally, it increases cost), and does not solve its oxidation stability problem. This clearly reveals the need to continue developing new salts and electrolytes for KIBs. The recently reported results on the use of non-flammable and fire-retardant electrolytes, i.e., TEP or TMP, should be the way forward in the search for safe KIBs.

Therefore, it is essential that upcoming studies focus on the overall composition of the electrode as well as the compatibility between electrodes and electrolytes, emphasizing the need to develop new materials and not directly transfer the knowledge gained from LIBs and NIBs.

**Figure 12:**
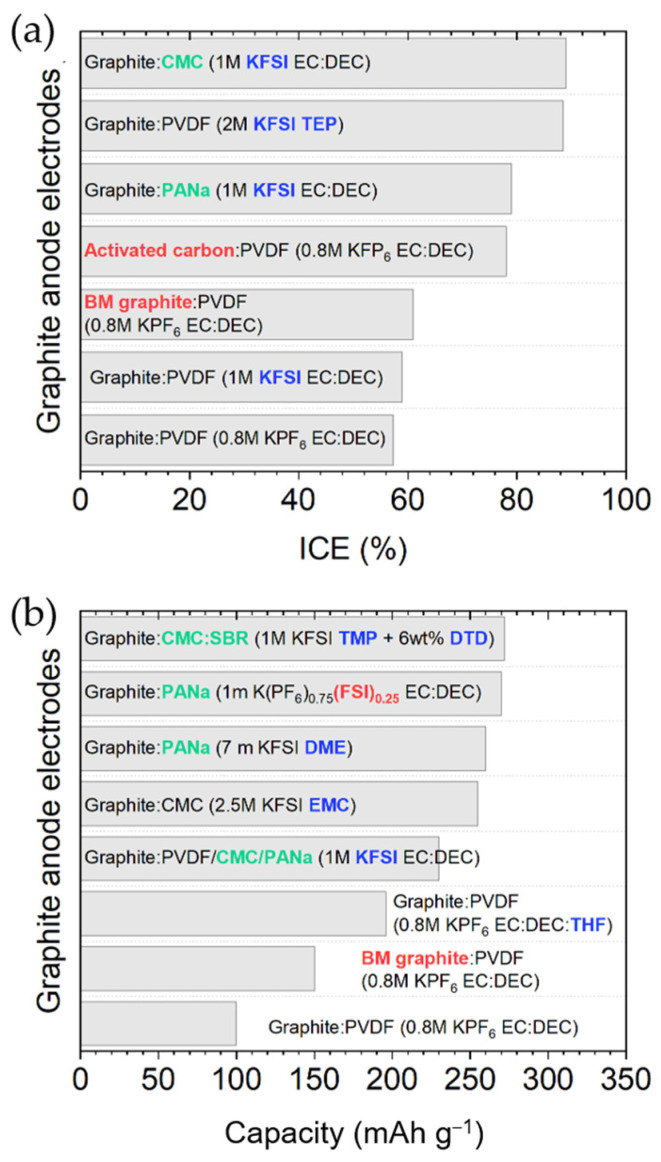
Comparison of (**a**) ICE and (**b**) capacities of graphite after 50–100 cycles* using different combinations of graphite (red), binders (green), and electrolytes (blue). Values gathered from Table 2. * The capacity of graphite: PVDF and graphite: CMC electrodes tested in 1 M KFSI in EC: DEC corresponds to a cyclability of only 20 and 8 cycles, respectively.

**Figure 13:**
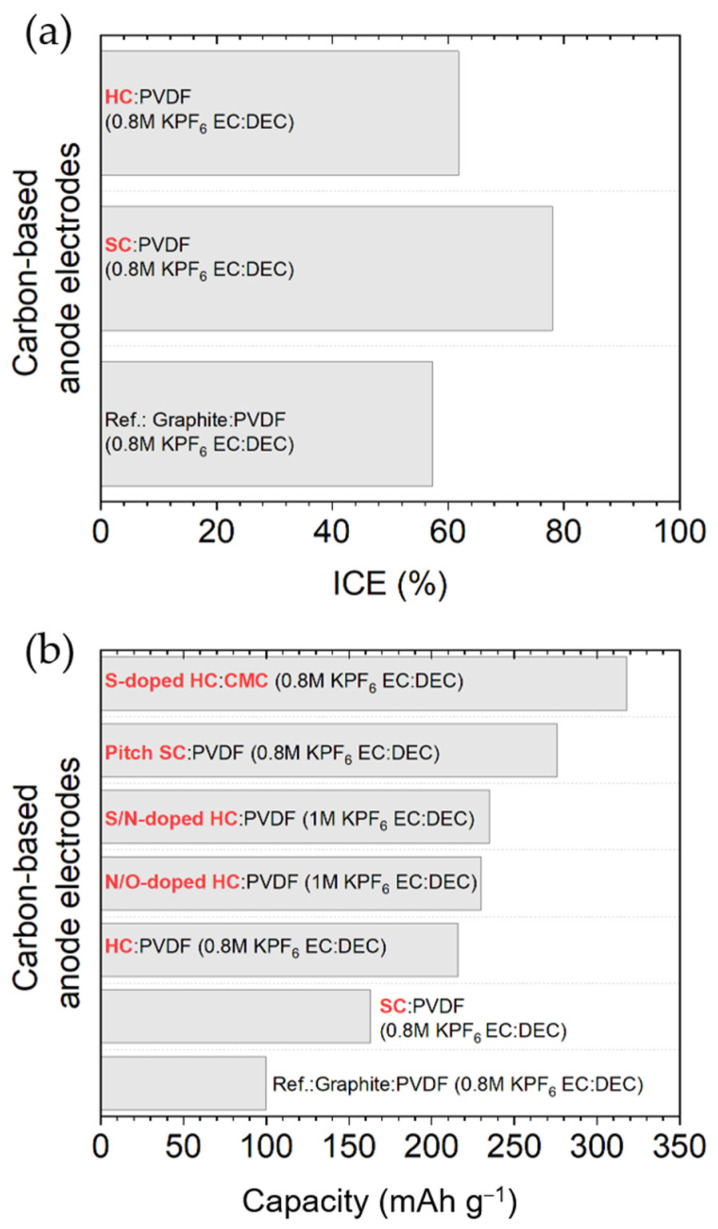
Comparison of (**a**) ICE and (**b**) capacities of SC or HC achieved after 50–100 cycles using different active material (red).

To identify which electrode composition and electrolyte chemistry are best for achieving carbon-based anode materials (e.g., graphite, soft and hard carbon) with high ICEs and capacities, the following analysis has been carried out. As values of reference, the parameters most identical to those used for commercial LIB technology were taken, i.e., graphite: PVDF electrode composition tested in 0.8 M KPF_6_ in EC:DEC electrolyte (see Table 2) [32]. The ICE and capacity were then compared, where only one parameter was modified (i.e., binder, electrolyte salt and solvent, or active material properties) and the exhibited ICE or capacity was higher than that of the reference system. Exceptionally, 0.8 M KFSI in EC:DEC can be considered as a second reference value for graphite (Figure 12a). Nevertheless, it is worth mentioning that it is impossible to accurately contrast the reported works on graphite-based and soft- and hard-carbon-based anode materials to understand their optimal properties and find the best electrode–electrolyte configuration because there is no standardized material, electrode composition, electrolyte chemistry, formulation, electrochemical cycling protocol, etc. Figure 12 and Figure 13 illustrate some critical properties for improving the ICE and capacity of graphite and soft- and hard-carbon electrodes, respectively.

For the particular case of graphite, the ICE (Figure 12a) is upgraded by adjusting the chemistry of the electrode and/or the electrolyte. Indeed, higher reversibility could be attained in the first cycle by controlling the particle size of the graphite (BM graphite) or replacing KFP_6_ with KFSI due to the formation of inorganic-rich SEI, as mentioned above. However, these results suggest that the most critical parameters to obtain a high ICE (>80%) correspond to the choice of the binder and/or the electrolyte solvent(s) that participate in the SEI formation. The best results (Figure 12a) have been achieved with aqueous-based CMC binder, KFSI salt, and EC:DEC carbonate solvents or with PVDF in a carbonate-free, non-flammable TEP-based solvent.

Regarding the capacity after 50–100 cycles (Figure 12b), a similar behavior is observed, i.e., the chemistry of the binder and electrolyte are decisive. The comparison indicates that the most critical component is again a good match between the binder and the electrolyte. In fact, the highest capacity (after 100 cycles) reported for graphite is achieved using CMC: SBR binder, TMP solvent, and DTD additive. Unfortunately, there is no direct correlation between high ICE and high capacity. For example, graphite: CMC electrodes tested in 1 M KFSI in EC:DEC exhibit the highest ICE (89%); however, the capacity they deliver (not included in Figure 12b) is only 230 mAh g^−1^. For their part, graphite: CMC: SBR electrodes tested in 1 M KFSI in TMP + 6 wt.% DTD exhibit a slightly lower ICE of 86.6%, but the highest capacity retention. Combining these results reveals that the optimal electrode and electrolyte chemistry should be based on aqueous binders (CMC, SBR) with KFIS and non-flammable electrolytes (TMP, or probably TEP). Additionally, the use of non-carbonate-based additives such as DTD seems to be crucial, where their concentration could be optimized. Therefore, further studies should be carried out to explore the possibility of developing superior graphite anode electrodes for KIBs.

A similar analysis has been performed with soft- and hard-carbon electrodes (values from Table 5), the other promising carbon-based anode materials. In terms of ICE (Figure 13a), there is not enough work reported to draw a precise conclusion. However, the soft-carbon material exhibits higher ICE in comparable systems, e.g., electrodes based on PVDF binder and tested in 0.8 M KPF_6_ in EC:DEC electrolyte. More findings could be found in terms of delivered capacities (Figure 13b). Both soft- and hard-carbon electrodes deliver higher capacities than graphite, which is easily explained by the adsorption phenomenon they experience in addition to the intercalation mechanism; this could be further increased by engineering the active material, such as changing the carbon precursor or resorting to heteroatom doping. In fact, heteroatom doping is one of the most promising strategies to enhance the specific capacity and long-term stability of hard carbon [186].

In summary, this analysis demonstrates the superior performance, with respect to certain electrochemical properties (such as ICE and capacity) evaluated here, of soft- and hard-carbon anodes relative to graphite for KIBs. Furthermore, it should be considered that soft- and hard-carbon anode electrodes were primarily tested with carbonate-based electrolytes and, as shown in the case of graphite, alternative chemistries, such as KFSI, glymes, or non-flammable electrolytes, could even lead to superior performances and be the path to follow.

Finally, although this review has focused on anode intercalation materials for practical applications of KIBs, the current performance evaluation is still insufficient and is mainly based on material-level and half-cell testing. Therefore, more attention needs to be paid to full-cell testing protocols from the laboratory to the device level, avoiding the use of K metal as a counter electrode, which could alter the results. In addition, it is advisable to go one step further, focusing on the cathode–anode degradation mechanism of the full cell as well as moving on to the cell device configuration.

## Figures and Tables

**Table 4:** Electrochemical performance of soft-carbon and hard-carbon anode materials in KIBs.

					Cyclability		Rate Capability	
Anode Material	Electrolyte	Binder	ICE	Initial Capacity (mAh g^−1^)@ Current Density (A g^−1^)	Capacity (mAh g^−1^)	Cycle Number@ Current Density (A g^−1^)	Capacity (mAh g^−1^)@ Current Density (A g^−1^)	Ref.
SC	0.8 M KPF_6_/EC:DEC	PVDF	~78%	~273@0.007	~163	50 cycles@0.56	140@1.4	[32]
SC hollow microrods	0.8 M KPF_6_/EC:DMC + 5 wt.% FEC	PTFE	~57%	~340@0.1	249	100 cycles@0.1	214@0.5	[110]
Pitch-derived SC	0.8 M KPF_6_/EC:DEC	CMC+SBR	~53%	296@0.028 200@0.28	276 183	50 cycles@0.028 1000 cycles@0.28	115@1.4	[111]
USC	0.8 M KPF_6_/EC:DEC	CMC	N/A	~1200@0.025 1080@0.1	280 137	50 cycles@0.025 2500 cycles@1	151@6.4	[113]
PVC-SC-800	0.8 M KPF_6_/EC:DEC	CMC	68.3%	~300@0.1	200	50 cycles@0.1	92@2	[114]
ONC-SC	0.8 M KPF_6_/EC:DEC	PVDF	55.7%	~323@0.05	106	500 cycles@1	99@2	[115]
HC microspheres	0.8 M KPF_6_/EC:DEC	PVDF	61.8%	262@0.028	216	100 cycles@0.028	136@1.4	[116]
CS-HC	2.5 M KFSI/TEP	CMC	87.3%	288@0.05 280@0.3	267 214	100 cycles@0.5 500 cycles@0.3	280@0.3	[119]
N-doped CS (chitin)	0.8 M KPF_6_/EC:DEC	PVDF	~88%	180@0.5	180	4000 cycles@0.5	154@20.2	[120]
N-doped HC (lignite)	1 M KPF_6_/EC:DEC	CMC	41.5%	392@0.1 N/A ^1^	314 125	100 cycles@0.1 1500 cycles@1	118@2	[121]
P-doped HC	0.8 M KPF_6_/EC:DEC	PVDF	N/A	160	~155	700 cycles@0.3	~175@0.5	[122]
S-doped HC	0.8 M KPF_6_/EC:DEC	CMC	35.1%	361@0.05	318	100 cycles@0.05	116@1.6	[123]
N/O-doped HC	1 M KPF_6_/EC:DEC	PVDF	25%	315@0.05 174@1.05	230 130	100 cycles@0.5 1100 cycles@1.05	118@3	[124]
S/O-doped HCMs ^2^	0.8 M KPF_6_/EC:DEC	PVDF	N/A	220@0.2 ~175@1	~200 ~110	200 cycles@0.2 2000 cycles@1	~175@1	[125]
S/N-doped HC	1 M KPF_6_/EC:DEC	PVDF	~35%	294@0.1 174@3	235 145	300 cycles@0.1 1200 cycles@3	174@3	[126]
HC/SC	0.8 M KPF_6_/EC:DEC	PVDF	N/A	N/A ^1^ ~166@1	185 101	100 cycles@0.1 500 cycles@1	121@3.2	[127]

^1^ N/A stands for not available. ^2^ HCMs = Hard carbon microspheres.

**Table 7:** Electrochemical performance of vanadium oxides (and derivatives) as anode materials in KIBs. N/A = not available.

					Cyclability		Rate Capability	
Anode Material	Electrolyte	Binder	ICE	Initial Capacity (mAh g^−1^)@ Current Density (A g^−1^)	Capacity (mAh g^−1^)	Cycle Number@ Current Density (A g^−1^)	Capacity (mAh g^−1^)@ Current Density (A g^−1^)	Ref.
K_0.23_V_2_O_5_	0.8 M KPF_6_/EC:DEC	PVDF	49.3%	404@0.02 ~265@0.1	122 97	150 cycles@0.02 100 cycles0.1	92@0.4	[170]
S-s. C@V_2_O_5_@CNFs ^1^	1 M KFS/EC:DMC	Free	44.9%	259@0.05 156@2	279 139	800 cycles@0.05 5000 cycles@2	156@2	[171]
VO@C	1 M KFS/EC:DEC	PVDF	35.5%	340@0.1 275@1	345 241	400 cycles@0.1 1000 cycles@1	136@10	[172]
K_2_V_3_O_8_	0.8 M KPF_6_/EC:DMC	CMC	~60%	282@0.05	84	180 cycles@0.1	103@0.5	[173]
SA-VO_2_	0.8 M KFSI/EC:DEC	PVDF	64.4%	290@0.05 205@0.5	288 177	50 cycles@0.05 500 cycles@0.5	141@2	[174]
VO_2_-V_2_O_5_/NC	0.8 M KPF_6_/EC:DMC:DEC	PVDF	N/A	N/A@0.1 ~540@1	501 256	120 cycles@0.1 1600 cycles@1	108@10	[175]
S-s. V_2_O_3_ NPs@PNCNFs ^1^	0.8 M KPF_6_/EC:DEC	Free	60%	215@0.05	206	500 cycles@0.05	134@1	[176]
HS-V_2_O_3_@C	3 M KFSI/DME	CMC	50.9%	~320@0.1	330	500 cycles@0.1	79@5	[177]
V_2_O_3_ NPs@C nanosheets	1 M KFSI/DME	PVDF	31%	~254@0.1 N/A@2	267 148	100 cycles@0.1 1800 cycles@2	116@5	[178]

^1^ S-s. = self-standing.

## Data Availability

No new data were created.
